# Hydrogen Activation on Zeolite Stabilized Ni–Mo Sulfide Clusters

**DOI:** 10.1021/jacsau.4c01088

**Published:** 2025-01-24

**Authors:** Rachit Khare, Roland Weindl, Sungmin Kim, Libor Kovarik, Andreas Jentys, Karsten Reuter, Johannes A. Lercher

**Affiliations:** a Department of Chemistry and Catalysis Research Center, Technical University of Munich, Garching 85748, Germany; b Institute for Integrated Catalysis, Pacific Northwest National Laboratory, Richland, Washington 99352, United States; c Theory Department, Fritz Haber Institute of the Max Planck Society, Berlin 14195, Germany

**Keywords:** transition metal sulfides, hydrogen activation, X-ray absorption spectroscopy, IR spectroscopy

## Abstract

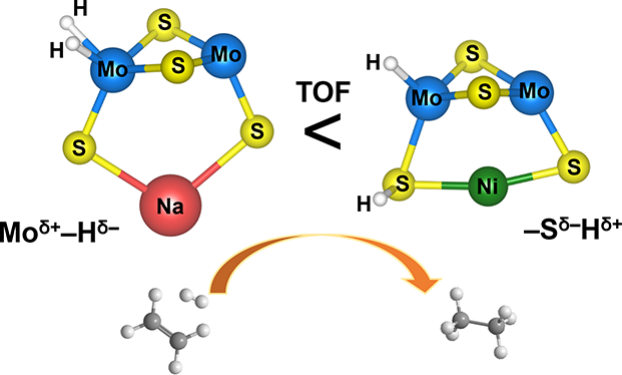

The activation of H_2_ on NaY-encapsulated Mo sulfide clusters is significantly influenced by the presence of Ni at ion exchange positions. Ni was incorporated by partially ion exchanging the NaY zeolite with Ni^2+^ cations. Mo(CO)_6_ vapors were subsequently deposited on the ion exchanged NiNaY zeolites followed by sulfidation in 10 vol % H_2_S/H_2_ at 673 K, leading to the formation of dimeric Mo_2_S_4_ clusters connected to Ni^2+^ via bridging S atoms. In contrast to the monometallic Mo sulfide clusters, which stabilize adsorbed hydrogen primarily as hydrides on Mo atoms, the bimetallic Ni–Mo sulfide clusters bind hydrogen also as sulfhydryl groups on the bridging sulfur atoms. The formation of sulfhydryl groups in Ni–Mo sulfide clusters is attributed to the lower electron density on the cluster due to coordination with more electronegative Ni^2+^. The ethene hydrogenation rate was significantly higher on the bimetallic Ni–Mo sulfide catalysts compared to monometallic Mo sulfide catalysts because the stabilization of atomic hydrogen as sulfhydryl groups opens a new hydrogenation pathway.

## Introduction

Transition metal sulfides (TMS) are important catalysts ubiquitously utilized for heteroatom removal from petroleum feedstock.^1−6^ These catalysts have been applied in many other processes such as upgrading of biomass-derived feedstock,^7−9^ water gas shift and reverse water gas shift reactions,^10,11^ CO_2_ conversion,^12−15^ and more recently even in electrocatalytic H_2_ evolution.^16−19^ Nature has also incorporated these sulfides as active sites in enzymes, like nitrogenase.^20^

Industrial TMS catalysts, typically based on the sulfides of Mo and W, are often promoted with the addition of other TMs, most prominently Ni and Co.^4,21−24^ These bimetallic TMS catalysts typically exceed the activity of monometallic counterparts. For example, CoMoS phase showed high activity for hydrodesulfurization (HDS) reactions.^25^ NiMoS and NiWS phases have also been reported to show superior hydrogenation activity.^26^ The active sites in the bimetallic TMS catalysts have been identified as coordinatively unsaturated sites (CUS) or sulfhydryl (−SH) groups, predominately present at the edges of the TMS slabs.^27−29^ The promoting effect of additional TMs has, thus, been explained by an increase in the concentration of CUS or sulfhydryl groups.^21,30−32^

Zeolites are conceptually interesting supports for achieving homogeneously distributed, well-defined TMS clusters. Several attempts have been made to stabilize TMS clusters within the micropores of zeolites.^33−36^ In a recent work, we demonstrated the homogeneous distribution of dimeric Mo_2_S_4_ and tetrameric Mo_4_S_4_ clusters encapsulated within NaY zeolites.^37^ These catalysts were synthesized using the chemical vapor deposition (CVD) of Mo(CO)_6_.^37^ Notably, the tetrameric Mo_4_S_4_ clusters exhibited electronic and structural characteristics similar to the cubane motifs found in the FeMo-cofactor of the nitrogenase enzyme.^37^

Encapsulation of TMS clusters containing multiple TMs within zeolite micropores has also been explored, although to a much lesser extent. For instance, Okomato et al. showed that Co_2_Mo_2_S_6_ clusters stabilized inside NaY zeolites exhibited improved activity for HDS.^38,39^ Similarly, De Bont et al. studied the coprecipitation of Co and Mo sulfide phases within NaY zeolites, but did not observe the formation of bimetallic clusters.^40^ In another study, Taniguchi et al. investigated the HDS activity of Ni–Mo sulfide clusters and demonstrated that Ni incorporation into the trimeric Mo_3_S_4_ clusters significantly improved their HDS activity.^41,42^

A significant number of reactions studied on TMS catalysts involve H_2_ as a reactant, prompting considerable effort into understanding the interactions and activation mechanism of H_2_ on these materials.^43−51^ It is widely accepted that H_2_ adsorbs dissociatively on these TMS catalysts, after which it stabilizes either as sulfhydryl groups (−S^δ−^H^δ+^) or as hydrides on the metal atoms (Mo^δ+^–H^δ−^).^52−57^ While previous studies have shown that molecular hydrogen is stabilized as sulfhydryl groups in bulk TMS phases,^52−54^ we have recently shown that the zeolite-encapsulated dimeric Mo_2_S_4_ and tetrameric Mo_4_S_4_ clusters stabilize hydrogen as hydrides on Mo atoms.^57^

We demonstrate here that hydrogen binding in Mo_2_S_4_ clusters varies in the presence of Ni introduced into the zeolite via partial ion exchange. The partially ion exchanged NiNaY zeolites were impregnated with Mo(CO)_6_ using CVD. Following sulfidation, we show that these carbonyl-containing catalyst precursors formed bimetallic Ni–Mo sulfide clusters within the zeolite micropores. The structure of these clusters has been elucidated by in situ X-ray absorption (XAS) and X-ray emission spectroscopy (XES), complemented by density functional theory (DFT) calculations and electron microscopy. Using infrared (IR) spectroscopy of adsorbed 2,4-dimethylpyridine (DMP), we reveal the dominant mode of hydrogen activation and stabilization on the bimetallic Ni–Mo sulfide clusters.

## Results and Discussion

The parent NaY zeolite (Zeolyst CBV 100; Si/Al ∼2.4; Al content ∼4400 μmol_Al_·g_cat_^–1^) was partially ion exchanged with Ni^2+^ ions to produce a series of NiNaY zeolites with varying nickel content. These NiNaY zeolites are designated as NiNaY(X), where “X” represents the Ni content (quantified by elemental analysis) expressed in μmol_Ni_·g_cat_^–1^. Following the ion exchange, the NiNaY zeolites were subjected to a sulfidation treatment in 10 vol % H_2_S/H_2_ at 673 K for 4 h. The resulting sulfided catalysts are referred to as NiS(X), where “X” corresponds to the Ni content (from elemental analysis) in μmol_Ni_·g_cat_^–1^.

The ion exchanged NiNaY zeolites were subsequently impregnated with varying amounts of Mo(CO)_6_ using CVD. The resulting Mo(CO)_6_-loaded precursors were sulfided in a 10 vol % H_2_S/H_2_ atmosphere at 673 K for 4 h. These sulfided catalysts are denoted as NiMoS(X,Y), where “X” and “Y” indicate the Ni and Mo contents (quantified from elemental analysis), respectively, expressed in μmol_metal_·g_cat_^–1^. In analogy to our previous study, the parent NaY zeolite (i.e., without the prior Ni^2+^ ion exchange) was then impregnated with varying amounts of Mo(CO)_6_ and sulfided under the same conditions.^37^ These catalysts are labeled as MoS(Y), where “Y” denotes the Mo content (from elemental analysis) in μmol_Mo_·g_cat_^–1^.

Supplementary Table S1 summarizes the Ni and Mo content in the catalyst samples investigated in this work. In the NiMoS zeolite series, derived from the same parent NiNaY zeolite, a systematic decrease in Ni content was observed as Mo loading increased. The higher Mo(CO)_6_ loading was achieved by extending the exposure of the NiNaY zeolites to Mo(CO)_6_ vapors. Prolonged exposure increased the likelihood of Ni removal through carbonylation, potentially forming volatile compounds like Ni(CO)_4_. Therefore, we attribute the slight decrease in Ni content with increasing Mo loading to the removal of Ni from its ion exchange positions via carbonylation during the CVD process.

Supplementary Figure S1 shows the IR spectra of pyridine (Py) adsorbed on the NiS(665) zeolite sample after in situ sulfidation. The spectra were recorded at varying pyridine partial pressures (*p*_*Py*_ ≈ 0.1–0.5 mbar) and after desorption under vacuum at ∼323 K. The IR spectra of pyridine adsorbed on the sulfided parent NaY zeolite and the sulfided monometallic MoS zeolite have been reported previously.^37^ The bands at ∼1441 cm^–1^ and ∼1450 cm^–1^, and those between 1573 and 1629 cm^–1^ are indicative of pyridine coordinated to Lewis acid sites (LAS) in the zeolite (denoted as Py-L).^58,59^ On the other hand, the bands at ∼1543 and ∼1639 cm^–1^ are characteristic of pyridine adsorbed on the Bro̷nsted acid sites (BAS) in the zeolites (denoted as Py-H^+^).^58,59^ The band at ∼1489 cm^–1^ is attributed to pyridine adsorbed on both BAS and LAS.^58,59^

Notably, on the sulfided parent NaY and the MoS zeolites, we only observed IR bands associated with Py-L.^37^ In contrast, the in situ sulfided NiS(665) zeolite sample additionally showed a small band at ∼1543 cm^–1^ (corresponding to Py-H^+^), indicating the presence of small concentrations of BAS in these materials (see Figure S1; Supporting Information). These BAS are likely formed either (i) during the ion exchange procedure or (ii) during the sulfidation treatment; for example, due to the dissociation of adsorbed H_2_S on the Ni^2+^ cations, leading to the formation of a Ni^2+^-coordinated SH^–^ groups (Ni^2+^–SH^–^) and a corresponding H^+^ on a neighboring framework oxygen atom.

The structure of NiNaY zeolite-encapsulated TMS clusters formed after the sulfidation treatment was elucidated using XAS at Mo and Ni K-edges. All spectra were recorded in situ following the sulfidation of the catalyst precursors in 10 vol % H_2_S/H_2_ at 673 K for 2 h. The Mo K-edge X-ray absorption near edge structures (XANES) of the representative NiMoS and MoS catalysts are presented in Supplementary Figure S2. The measured XANES clearly indicate the formation of Mo–S bonds during the sulfidation process.

**Figure 1:**
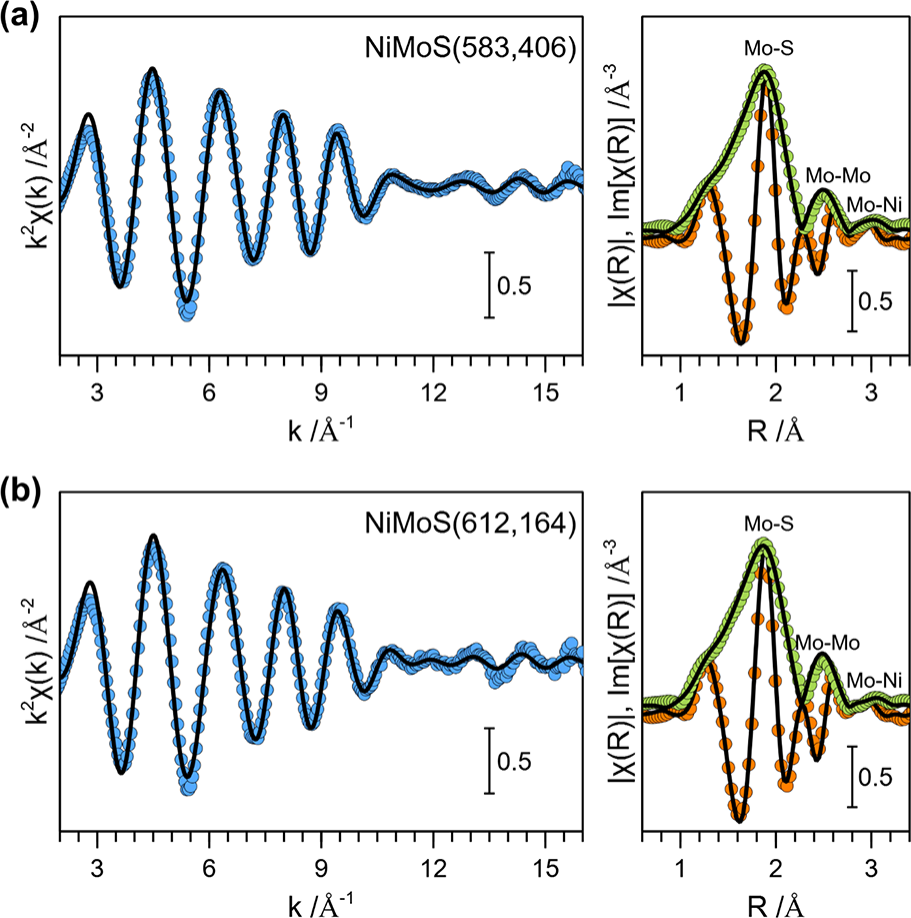
Mo K-edge *k*^2^-weighted EXAFS (left panel) and FT-EXAFS (right panel) of NiMoS(583,406) and NiMoS(612,164) catalysts. Experimental data are shown as closed symbols, while the corresponding fits are shown as solid black lines. All spectra were measured in situ following the sulfidation of the catalyst precursors in 10 vol % H_2_S/H_2_ atmosphere at 673 K for 2 h.

Figure 1 shows the Mo K-edge (20000 eV) extended X-ray absorption fine structure (EXAFS) of two NiMoS catalysts with different Ni/Mo ratios. The EXAFS of a representative MoS(440) catalyst is presented in Supplementary Figure S3. EXAFS fitting was performed using Mo–S and Mo–Mo single-scattering paths, with bond distances (*d*) of *d*_*Mo*–*S*_ ≈ 2.39 Å and *d*_*Mo*–*Mo*_ ≈ 2.79 Å, which correspond to the first and second coordination shells, respectively. Additionally, a Mo–Ni single-scattering path was included with *d*_*Mo*–*Ni*_ ≈ 3.30 Å to improve the fit quality. The fitted EXAFS parameters are summarized in Table 1. For a detailed overview of the fitting results, refer to Supplementary Table S2.

**Table 1:** Mo K-Edge EXAFS Fitting Parameters: Coordination Numbers (**CN**), Interatomic Distances (**d** /Å), and the Debye–Waller Factors (**σ**^2^ /10^–3^·Å^2^) for MoS(440) and NiMoS Catalysts with Different Ni/Mo Ratios

**catalyst**	Mo–S			Mo–Mo			Mo–Ni		
	**CN**	**d**	**σ**^**2**^	**CN**	**d**	**σ**^**2**^	**CN**	**d**	**σ**^**2**^
MoS(440)	3.8	2.42	6.4	1.0	2.77	8.9^a^			
NiMoS(583,406)	3.8	2.38	7.5	0.9	2.80	6.3^a^	0.39	3.29	6^a^
NiMoS(612,164)	4.2	2.38	8.7	1.1	2.79	6.3^a^	0.23	3.30	6^a^
Mo_*x*_S_*y*_/NaY	3.9^b^	2.42^b^		1.3^b^	2.77^b^				

a This parameter was fixed during the fit.

b Data from Weindl et al.^37^

As expected, the Mo–S and Mo–Mo CNs (*CN*_*Mo*–*S*_ ≈ 3.8 and *CN*_*Mo*–*Mo*_ ≈ 1.0) and the corresponding interatomic distances (*d*_*Mo*–*S*_ ≈ 2.42 Å and *d*_*Mo*–*S*_ ≈ 2.77 Å) for MoS(440) zeolite were similar to those previously reported for the sulfided Mo_*x*_S_*y*_/NaY zeolite sample (also presented in Table 1).^37^ Based on the structural parameters derived from EXAFS and aided by DFT calculations, we had concluded that dimeric Mo_2_S_4_ clusters are formed within the micropores of Mo_*x*_S_*y*_/NaY zeolites.^37^

Notably, the Mo–S and Mo–Mo CNs (*CN*_*Mo*–*S*_ ≈ 3.8–4.2 and *CN*_*Mo*–*Mo*_ ≈ 0.9–1.1, respectively) and interatomic distances (*d*_*Mo*–*S*_ ≈ 2.39 Å and *d*_*Mo*–*S*_ ≈ 2.80 Å, respectively) in the sulfided NiMoS zeolites were similar to those obtained for MoS(440). These similarities in the structural parameters suggest that the coordination environment around the Mo atoms in the bimetallic NiMoS catalysts closely resembles that of the dimeric Mo_2_S_4_ clusters. Therefore, based on the EXAFS analysis, we conclude that Mo_2_S_4_-like dimeric clusters are likely formed within the micropores of Mo(CO)_6_-impregnated NiNaY zeolites during the sulfidation treatment. It is important to mention here that in the proposed dimeric Mo_2_S_4_-like clusters, the theoretical *CN*_*Mo*–*S*_ is three.^37^ However, the fitted *CN*_*Mo*–*S*_ for the NiMoS and MoS catalysts was closer to four. The slightly higher Mo–S coordination is likely due to the adsorption or dissociation of H_2_S on the Mo atoms following the sulfidation treatment, as the XAS measurements were performed in situ under a 10 vol % H_2_S/H_2_ flow.

Remarkably, in the bimetallic NiMoS zeolites, an additional contribution from a Mo–Ni single-scattering path (with *d*_*Mo*–*Ni*_ ≈ 3.29 Å) was evident. This additional feature indicates the presence of Ni atoms at a distance of ∼3.3 Å from the Mo atoms. The relatively large Mo–Ni interatomic distance suggests that the Mo and Ni atoms are connected through Mo–S–Ni linkages, rather than a direct covalent Mo–Ni bond. Therefore, any direct interaction between Mo and Ni atoms is likely absent in these bimetallic catalysts. Furthermore, the relatively low Mo–Ni coordination (*CN*_*Mo*–*Ni*_ ≈ 0.23–0.39) indicates that not all Mo atoms are connected to Ni atoms *via* the bridging Mo–S–Ni bond.

The fit quality improved further with the inclusion of a Mo–O single-scattering path with *d*_*Mo*–*O*_ ≈ 1.61 Å (see Table S2; Supporting Information). The presence of oxygen atoms at this short distance suggests partial coordination of the cluster Mo atoms to the framework oxygen. It is also worth noting that the theoretical first-shell Mo–O distance in bulk Mo oxide material is ∼1.66 Å. Therefore, the observed Mo–O scattering at ∼1.61 Å could also be attributed to the formation of small amounts of bulk Mo oxide phase in the zeolites during the XAS measurements, possibly due to the presence of H_2_O or O_2_ impurities in the gas stream. Nevertheless, the low value of *CN*_*Mo*–*O*_ (less than 0.4) suggests that the formation of bulk Mo oxide phase, if any, was not significant in the synthesized NiMoS and MoS catalysts.

Next, to elucidate the coordination environment around the Ni atoms in the bimetallic NiMoS catalysts, we performed XAS measurements at the Ni K-edge (8333 eV). Again, all spectra were recorded in situ following the sulfidation of the catalyst precursors in 10 vol % H_2_S/H_2_ at 673 K for at least 2 h. Supplementary Figure S4 shows the Ni K-edge XANES of bimetallic NiMoS and monometallic NiS zeolites, clearly indicating the formation of a Ni sulfide phase in these samples.

**Figure 2:**
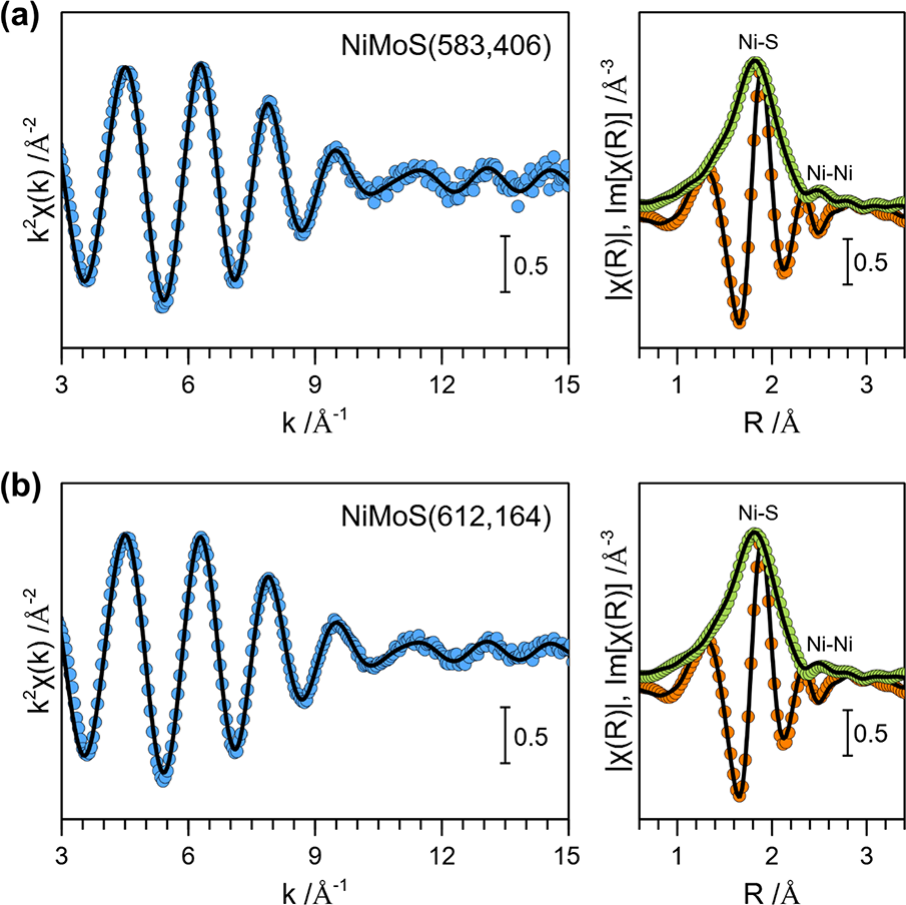
Ni K-edge *k*^2^-weighted EXAFS (left panel) and FT-EXAFS (right panel) of NiMoS(583,406) and NiMoS(612,164) catalysts. Experimental data are shown as closed symbols, while the corresponding fits are shown as solid black lines. All spectra were measured in situ following the sulfidation of the catalyst precursors in 10 vol % H_2_S/H_2_ atmosphere at 673 K for 2 h.

Figure 2 presents the Ni K-edge EXAFS and FT-EXAFS of NiMoS(583,406) and NiMoS(612,164) catalysts. For comparison, the EXAFS and FT-EXAFS of sulfided parent NiNaY(665) zeolite, referred to as NiS(665), are provided in Supplementary Figure S5. The EXAFS fitting was carried out using Ni–S and Ni–Ni single-scattering paths with *d*_*Ni*–*S*_ ≈ 2.23 Å and *d*_*Ni*–*Ni*_ ≈ 2.52 Å, respectively. These paths represent the first-shell Ni–S and second-shell Ni–Ni coordination, respectively. Additionally, a third-shell Ni–S_2_ single-scattering path with *d*_*Ni*–*S*_2__ ≈ 3.59 Å was also included in some cases to improve the fit quality. The EXAFS fitting parameters are summarized in Table 2. For detailed fitting results, see Supplementary Table S3.

**Table 2:** Ni K-Edge EXAFS Fitting Parameters: Coordination Numbers (**CN**), Interatomic Distances (**d** /Å), and the Debye–Waller Factors (**σ**^2^ /10^–3^·Å^2^) for NiS(665) and NiMoS Catalysts with Different Ni/Mo Ratios

**catalyst**	Ni–S			Ni–Ni			Ni–S_2_		
	**CN**	**d**	**σ**^**2**^	**CN**	**d**	**σ**^**2**^	**CN**	**d**	**σ**^**2**^
NiS(665)	3.6	2.18	4.0	1.0	2.50	6			
NiMoS(583,406)	3.9	2.23	5.6	1.1	2.54	4.6	0.27	3.53	6^a^
NiMoS(612,164)	4.2	2.23	6.3	1.1	2.53	5.4	0.34	3.50	6^a^
Ni_3_S_2_	4^b^	2.25^b^		4^b^	2.50^b^		4^b^	3.67^b^	
NiS	5^b^	2.25^b^		2^b^	2.50^b^		1^b^	3.67^b^	

a This parameter was fixed during the fit.

b Estimated from the theoretical Ni_3_S_2_ and NiS structures.

We note that the first- and second-shell Ni–S and Ni–Ni CNs (*CN*_*Ni*–*S*_ ≈ 3.6–4.2 and *CN*_*Ni*–*S*_ ≈ 3.6–4.2, respectively) and the corresponding interatomic distances (*d*_*Ni*–*S*_ ≈ 2.18–2.23 Å and *d*_*Ni*–*S*_ ≈ 2.50–2.54 Å, respectively) in both NiMoS catalysts and the parent NiS(665) zeolites were similar. Furthermore, the first-shell Ni–S CNs and distances in these materials were similar to the theoretical values for bulk Ni sulfide phases (for example, *CN*_*Ni*–*S*_ = 4 and *d*_*Ni*–*S*_ = 2.25 Å for Ni_3_S_2_ and *CN*_*Ni*–*S*_ = 5 and *d*_*Ni*–*S*_ = 2.25 Å for NiS, respectively; see Table 2). These structural similarities in EXAFS, together with the XANES, indicate the presence of bulk Ni sulfide phase in these NiMoS and NiS catalysts.

Interestingly, the Ni–Ni CNs in the NiS and NiMoS catalysts (*CN*_*Ni*–*Ni*_ ≈ 1.0–1.1) were significantly lower than the theoretical values expected for bulk Ni sulfide (e.g., *CN*_*Ni*–*Ni*_ = 4 in Ni_3_S_2_ and *CN*_*Ni*–*Ni*_ = 2 in NiS, respectively; see Table 2). In addition, the third-shell Ni–S_2_ CN was also significantly less intense in all NiMoS samples that we investigated with XAS. The low *CN*_*Ni*–*Ni*_ suggests that, in addition to the formation of bulk Ni sulfide, a significant portion of Ni might exist as isolated Ni species (which lack Ni–Ni coordination, i.e., *CN*_*Ni*–*Ni*_ = 0). The low *CN*_*Ni*–*S*_2__ also suggests that the bulk Ni sulfide phase formed within the zeolite micropores is primarily comprised of relatively small Ni sulfide nanoclusters.

Finally, the average first-shell Ni–S interatomic distance in the synthesized NiMoS catalysts (*d*_*Ni*–*S*_ ≈ 2.18–2.23 Å) was slightly shorter than the theoretical first-shell Ni–S interatomic distances in bulk Ni sulfide (e.g., *d*_*Ni*–*S*_ = 2.25 Å for both Ni_3_S_2_ and NiS; see Table 2). This slightly shortened average Ni–S distance supports the formation of relatively small Ni sulfide nanoclusters with a higher proportion of terminal sulfur atoms, as the Ni–S distance is likely to be shorter for the terminal sulfur atoms compared to the bridging S atoms.

**Figure 3:**
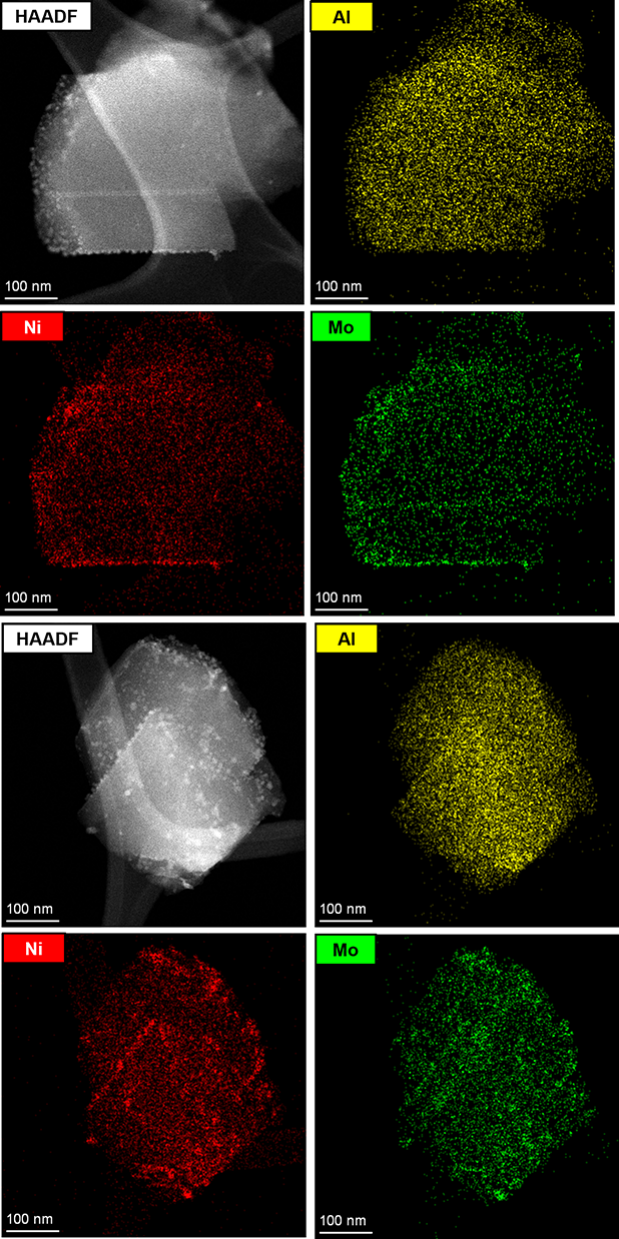
HAADF-TEM micrographs and EDXS elemental mappings of Al, Ni and Mo of two distinct crystallites of a representative NiMoS(583,406) catalyst sample.

Figure 3 presents the high-angle annular dark field-transmission electron microscopy (HAADF-TEM) micrographs and the energy dispersive X-ray spectroscopy (EDXS) elemental mapping of two distinct crystallites of a representative NiMoS(583,406) zeolite sample. The TEM images clearly indicate the formation of metal sulfide nanoparticles within the micropores of the zeolite crystallites as well as on the external surface. Also, the EDXS mapping of Ni exhibited localized regions of high Ni concentration. While regions of high Mo concentration were also detected, they were significantly less prominent. Therefore, we conclude that Mo is mostly homogeneously distributed throughout the zeolite crystallites. We recall here that the formation of a bulk Mo sulfide slabs (on the external surface) was also not observed for the monometallic MoS catalysts.^37^

The EDXS mapping of Ni indicated that, apart from formation of NiMoS_*x*_ nanoparticles within the larger micropores of faujasite and on the external surface, a significant fraction of Ni is also distributed homogeneously within the zeolite. Based on the Ni K-edge EXAFS and HAADF-TEM analysis, we conclude that Ni in the bimetallic NiMoS zeolites exists in two distinct forms: (i) bulk Ni(Mo) sulfide nanoparticles primarily located on the external surface of the zeolite crystallites, and (ii) isolated Ni^2+^ species within the zeolite micropores, mostly occupying the ion exchange sites.

We also note here that a lack of significant Ni–O coordination and high Ni–S coordination numbers (*CN*_*Ni*–*S*_ ≈ 3.6–4.2; Table 2) in the Ni K-edge EXAFS suggests that these isolated Ni^2+^ species are predominantly coordinated to sulfur atoms, and not to framework oxygen atoms. This Ni–S coordination likely results from adsorbed (or dissociated) H_2_S molecules interacting with the Ni^2+^ cations. H_2_S adsorption would displace Ni^2+^ from its ion exchange position, similar to the way hydration of Ni-containing zeolites displaces Ni^2+^ from its ion exchange sites by forming [Ni(H_2_O)_*x*_]^2+^ complexes.

Hypothesizing that Ni in the bulk sulfide phase exists as either Ni_3_S_2_ or NiS (with a theoretical *CN*_*Ni*–*Ni*_ of two or four, respectively) alongside isolated Ni^2+^ species (with a theoretical *CN*_*Ni*–*Ni*_ of zero), we can estimate the distribution of Ni between these two phases in the bimetallic NiMoS catalysts. Based on the second-shell Ni–Ni CNs obtained for the NiMoS catalysts (*CN*_*Ni*–*S*_ ≈ 1.0–1.1; Table 2), we estimate that approximately 35 ± 10% of Ni is present as bulk Ni sulfide phase.

**Figure 4:**
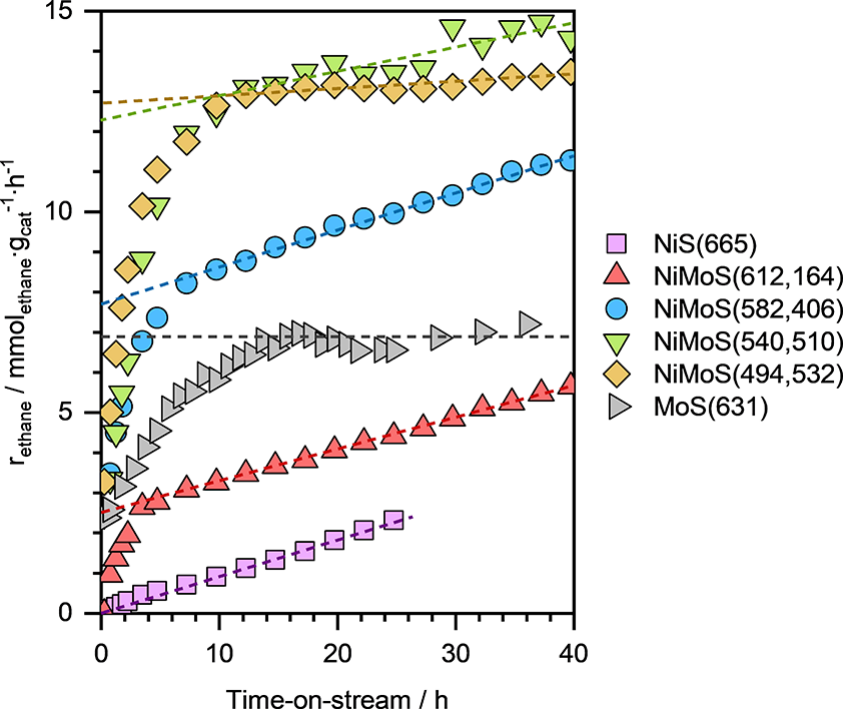
Ethane formation rates as a function of TOS during ethene hydrogenation on various NiMoS catalysts with different Ni/Mo ratios. The ethane formation rates on MoS(631) and NiS(665), representative of monometallic catalysts, are also shown. The dashed lines are linear regression fits to data at TOS > 15 h. Reaction conditions: *T* ≈ 473 K, *p*_*H*_2__ ≈ 95 kPa, *p*_*ethene*_ ≈ 5 kPa, and space-velocity ≈ 30–50 mmol_ethene_·g_cat_^–1^·h^–1^.

Next, we evaluated the hydrogenation activity of the synthesized NiS, MoS, and NiMoS catalysts prototypically by measuring the rate of ethene hydrogenation (at *T* ≈ 473 K, *p*_*ethene*_ ≈ 5 kPa, and *p*_*H*_2__ ≈ 95 kPa). Figure 4 presents the ethane formation rates (*r*_*ethane*_) on various NiMoS catalysts with different Ni/Mo ratios as a function of time-on-stream (TOS). For comparison, the *r*_*ethane*_ values, as a function of TOS, for representative monometallic MoS(631) and NiS(665) catalysts are also presented.

The monometallic MoS(631) catalyst exhibited stable hydrogenation activity for several hours, with an ethane formation rate of ∼6.9 mmol_ethane_·g_cat_^–1^. This aligns well with the previously reported ethene hydrogenation activity on Mo_*x*_S_*y*_/NaY catalysts under similar reaction conditions.^37,57^ Notably, the stable hydrogenation activity of the MoS catalysts was preceded by an induction period, marked by a logarithmic increase in ethene hydrogenation rates. This induction period is hypothesized to be caused by deblocking of the active sites by either (i) reversibly adsorbed H_2_S molecules on Mo atoms after the sulfidation, or (ii) additional terminal sulfur atoms bound to the Mo atoms of the clusters. We also recall here that the fitted *CN*_*Mo*–*S*_ for both NiMoS and MoS catalysts (*CN*_*Mo*–*S*_ ≈ 3.8–4.2; see Table 1) was higher than the theoretically predicted *CN*_*Mo*–*S*_ of three for the dimeric Mo_2_S_4_ clusters. The gradual desorption of H_2_S and/or the removal of terminal sulfur atoms as H_2_S, due to their reaction with molecular H_2_ in the reactant stream, likely exposes the coordinatively unsaturated Mo atoms that are the active sites for ethene hydrogenation reaction.

Similar to the MoS(631) catalyst sample, the monometallic NiS(665) catalyst also exhibited a very long induction period during ethene hydrogenation under same reaction conditions. Consequently, the ethane formation rate on NiS(665) increased almost linearly with the TOS (see Figure 4). This gradual increase in *r*_*ethane*_ is tentatively attributed to the slow removal of terminal sulfur atoms from the isolated Ni sulfide species, progressively exposing the coordinatively unsaturated Ni that are likely the active sites for ethene hydrogenation.

Remarkably, during ethene hydrogenation on bimetallic NiMoS catalysts under the same reaction conditions, we observed both an induction period marked by a rapid (logarithmic) increase in the hydrogenation activity, and a prolonged induction period with linearly increasing ethane formation rates. To deconvolute these contributions, we employed a linear regression model to fit the *r*_*ethane*_ at TOS > 15 h (denoted as dashed lines in Figure 4). The y-intercept of the fitted line was then used to estimate the “*effective*” *r*_*ethane*_ specifically attributable to the Mo containing active sites of the bimetallic NiMoS catalysts.

**Figure 5:**
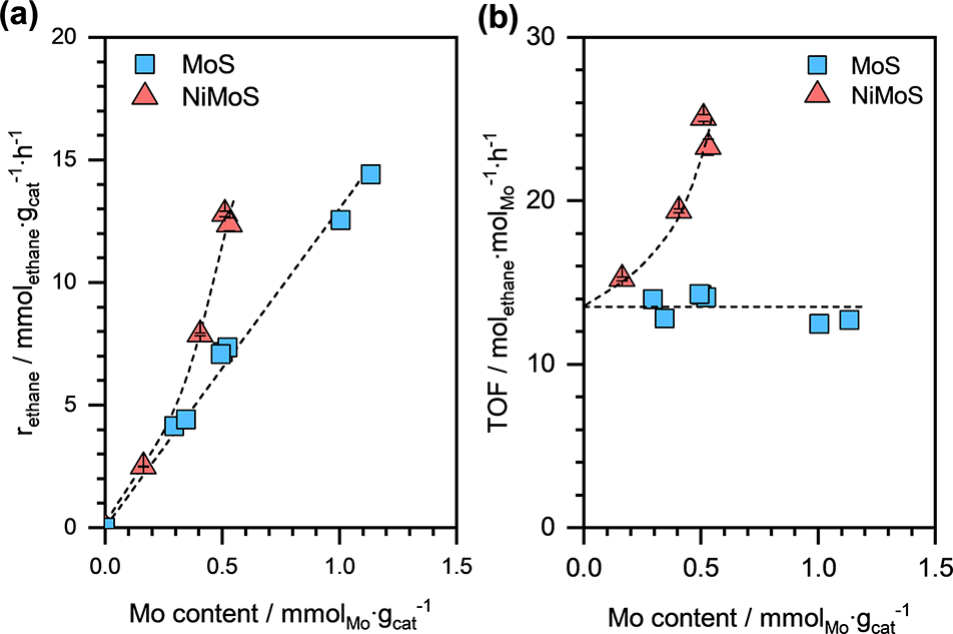
(a) Steady-state ethane formation rates and (b) ethene hydrogenation TOFs on monometallic MoS^37,57^ and bimetallic NiMoS catalysts as a function of net Mo content. The dashed lines are visual guides. Reaction conditions: *T* ≈ 473 K, *p*_*H*_2__ ≈ 95 kPa, *p*_*ethene*_ ≈ 5 kPa, and space-velocity ≈ 30–50 mmol_ethene_·g_cat_^–1^·h^–1^. Data for the monometallic MoS catalysts is adapted from ref (37). Available under a CC BY-NC-ND 4.0 license. Copyright 2021 Weindl et al.

Figure 5(a) presents the calculated “*effective*” *r*_*ethane*_ for a series of NiMoS catalysts, with same parent NiNaY zeolite, as a function of their Mo content. For comparison, the steady-state *r*_*ethane*_ on the monometallic MoS catalysts with varying Mo content are also shown.^37,57^ As previously reported, the *r*_*ethane*_ on MoS catalysts increases linearly with increasing Mo loading, which corresponds to a constant ethene hydrogenation turnover frequency (TOF) of approximately 13.4 mol_ethane_·mol_Mo_^–1^·h^–1^ or 6.7 mol_ethane_·mol_cluster_^–1^·h^–1^ for the dimeric Mo_2_S_4_ clusters (also presented in Figure 5(b)).

In contrast to the monometallic MoS catalysts, the “*effective*” *r*_*ethane*_ on the bimetallic NiMoS catalysts increased exponentially with increasing Mo loading (see Figure 5(a)). Also, the corresponding ethene hydrogenation TOFs for the bimetallic NiMoS catalysts were consistently higher than those for the monometallic MoS catalysts and increased systematically with increasing Mo content (see Figure 5(b)). Overall, these trends suggest a synergistic effect between Ni and Mo species for ethene hydrogenation reaction on the bimetallic NiMoS catalysts.

We recall that the structural parameters derived from the Mo K-edge EXAFS (see Table 1) indicated the formation of homogeneously distributed dimeric Mo_2_S_4_-like clusters in the bimetallic NiMoS catalysts. Furthermore, using the Ni–Ni CNs (obtained from the Ni K-edge EXAFS; see Table 2), we estimated that approximately 35 ± 10% of the Ni exists as bulk Ni sulfide nanoclusters, while the remaining 65 ± 10% Ni exists as isolated Ni species within the zeolite micropores. Assuming a stochastic distribution of the dimeric Mo sulfide clusters and the isolated Ni species within the zeolite micropores, we calculated the likelihood of a faujasite supercage containing these species (reported in Table S4; Supporting Information).

We also recall here that the Mo–Ni CNs in the bimetallic NiMoS(612,164) and NiMoS(583,406) catalyst samples were estimated to be ∼0.23 and ∼0.39, respectively (see Table 1). The probability of isolated Ni species and dimeric Mo species coexisting within the same faujasite supercage is estimated to be ∼0.06 for NiMoS(612,164) and ∼0.15 for NiMoS(583,406) catalyst (see Table S4; Supporting Information). Therefore, the *CN*_*Mo*–*Ni*_ increased systematically with the stochastic probability of Ni and Mo species coexisting within the same faujasite supercage. This correlation further suggests the formation of bimetallic NiMo sulfide clusters and a synergistic effect between Ni and Mo species, which enhances the ethene hydrogenation activity in the bimetallic NiMoS catalysts.

Based on these observations and correlations, we postulate that the coexistence of both isolated Ni species and dimeric Mo species within the same faujasite supercage leads to the formation of bimetallic NiMo_2_S_4_-like clusters that are characterized by bridging sulfur atoms between the Ni and Mo atoms. These bimetallic clusters are likely formed through the interaction of the dimeric Mo_2_S_4_ clusters (formed during the sulfidation treatment) with the isolated Ni^2+^ cations present at ion exchange positions within the same faujasite supercage.

**Figure 6:**
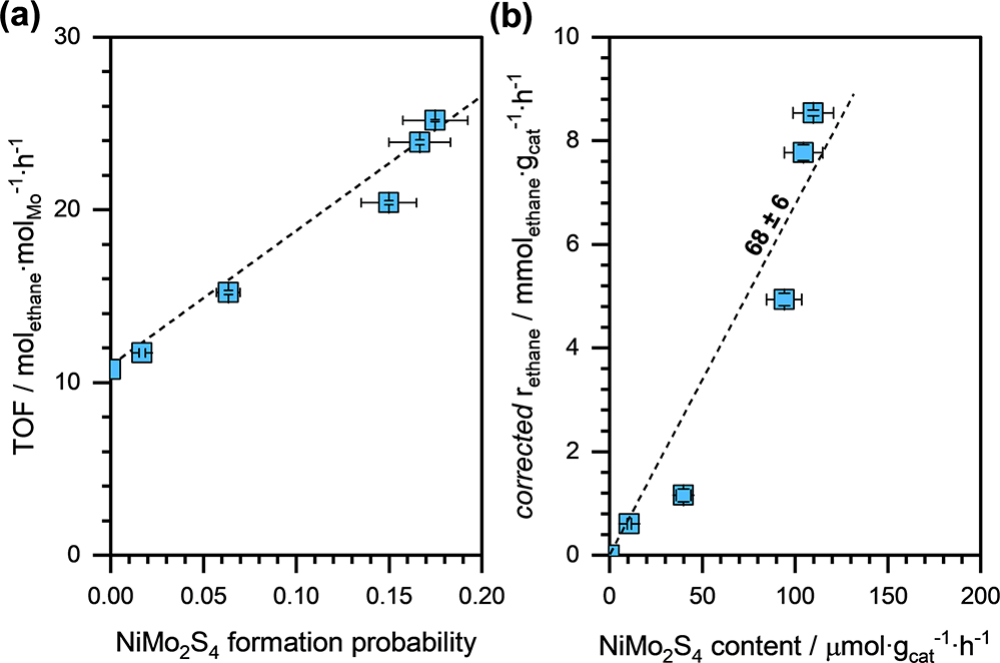
(a) Ethene hydrogenation TOF as a function of the stochastically predicted probability of NiMo_2_S_4_ cluster formation within the faujasite supercage of NiMoS catalysts. (b) “*corrected*” ethane formation rates as a function of NiMo_2_S_4_ cluster content in the bimetallic NiMoS catalysts. The dashed lines are linear regression fits. Reaction conditions: *T* ≈ 473 K, *p*_*H*_2__ ≈ 95 kPa, *p*_*ethene*_ ≈ 5 kPa, and space-velocity ≈ 30–50 mmol_ethene_·g_cat_^–1^·h^–1^. The stochastic distribution was calculated assuming that the bulk Ni sulfide phase is Ni_3_S_2_.

Figure 6(a) shows the ethene hydrogenation TOF, normalized to the total Mo content, as a function of the stochastically predicted probability of NiMo_2_S_4_ cluster formation within a faujasite supercage. We can clearly see that the TOF increased almost linearly with the stochastically predicted probability of NiMo_2_S_4_ cluster formation, indicating that the intrinsic ethene hydrogenation rates on the bimetallic NiMo_2_S_4_ clusters are significantly higher than that on the monometallic Mo_2_S_4_ clusters.

Using the theoretical concentration of faujasite supercages in the Y zeolite (equal to ∼628 μmol_supercage_·g_cat_^–1^), we calculated the (stochastically predicted) concentration of NiMo_2_S_4_ clusters in the bimetallic NiMoS catalysts. Then, by subtracting the amount of Mo present as NiMo_2_S_4_ from the total Mo content, we estimated the concentration of monometallic Mo_2_S_4_ clusters present in the bimetallic catalysts. Finally, assuming that the overall ethene hydrogenation rate on the bimetallic NiMoS catalysts is a linear combination of the individual ethene hydrogenation rates on the NiMo_2_S_4_ and Mo_2_S_4_ clusters, we estimated the ethane formation rate specifically attributable to the NiMo_2_S_4_ clusters (referred to as the “*corrected*” *r*_*ethane*_). For this calculation, we assumed that the *r*_*ethane*_ on Mo_2_S_4_ clusters is equal to 13 mol_ethane_·mol_Mo_^–1^·h^–1^.

Figure 6(b) shows the “*corrected*” *r*_*ethane*_ (normalized to the catalyst weight) as a function of stochastically predicted NiMo_2_S_4_ content in the NiMoS catalysts. We can see that the “*corrected*” *r*_*ethane*_ increased monotonically with increasing NiMo_2_S_4_ content. From a linear regression analysis, we estimated the ethene hydrogenation TOF on NiMo_2_S_4_ clusters to be approximately 68 ± 6 mol_ethane_·mol_cluster_^–1^·h^–1^, i.e., about 1 order of magnitude higher than the TOF on the monometallic Mo_2_S_4_ clusters (∼6.7 mol_ethane_·mol_cluster_^–1^·h^–1^).

**Figure 7:**
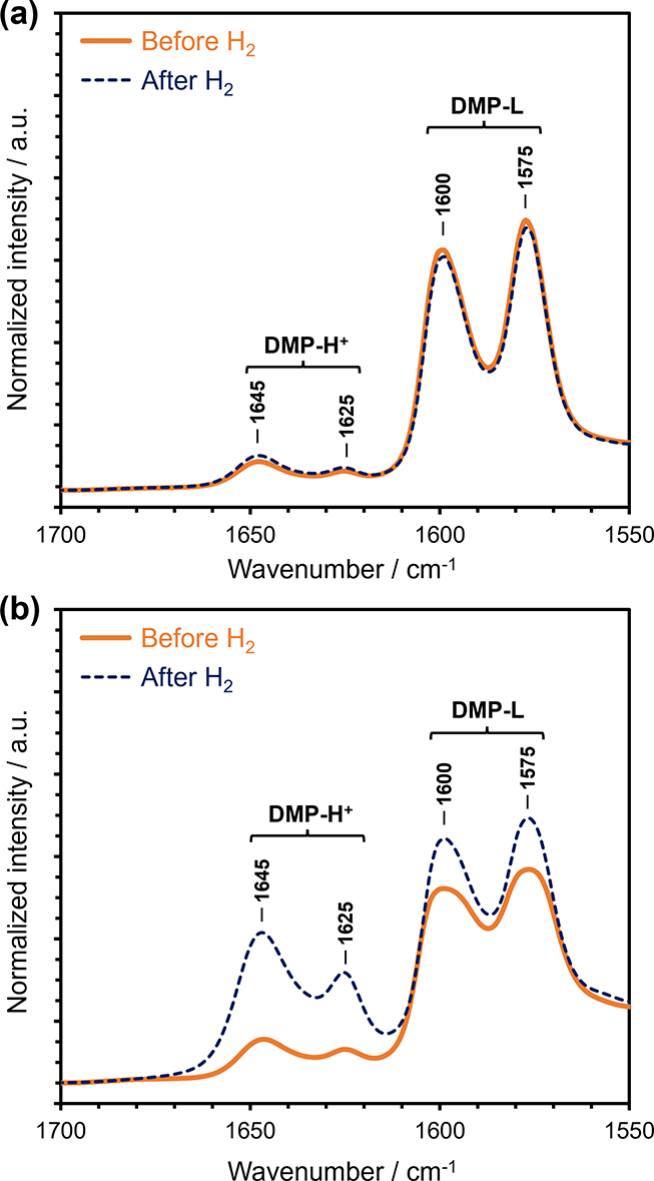
IR spectra of DMP adsorbed on a (a) NiS(665) (top panel) and (b) NiMoS(494,532) (bottom panel) catalysts, before (solid orange lines) and after (dashed blue lines) admission of H_2_. Spectra were collected at 323 K after outgassing for 30 min. All spectra are normalized to catalyst wafer thickness and mass. Prior to the measurements, the samples were sulfided in situ in a 10 vol % H_2_S/H_2_ at 623 K for 2 h.

Next, to elucidate the reason behind enhanced hydrogenation activity of bimetallic NiMo_2_S_4_ clusters compared to the monometallic Mo_2_S_4_ clusters, we performed IR spectroscopy of adsorbed DMP. Figure 7 shows the IR spectra of DMP adsorbed on the presulfided NiS(665) and NiMoS(494,532) catalysts. The IR spectra of DMP adsorbed on a sulfided monometallic MoS catalyst sample has been reported elsewhere.^57^ All spectra were recorded at 323 K before and after the admission of H_2_ and subsequent outgassing.

Notably, all catalyst samples displayed characteristic IR bands at ∼1575 and ∼1600 cm^–1^, assigned to DMP coordinated to LAS (denoted as DMP-L),^60^ and at ∼1625 and ∼1645 cm^–1^, which are assigned to protonated DMP (denoted as DMP-H^+^).^61^ In the sulfided NiS and the MoS catalysts (see Figure 7(a) and ref (57), respectively), the intensity of IR bands associated with DMP-H^+^ was low. Furthermore, the intensity of this band did not increase after the admission of H_2_. Therefore, we conclude that the formation of sulfhydryl groups in both monometallic MoS and NiS catalysts is negligibly low.^57^ On the other hand, in the sulfided NiMoS catalyst, the intensity of DMP-H^+^ bands increased significantly after the exposure of the catalyst to H_2_ (see Figure 7(b)). These pronounced bands clearly indicate that the Ni–S–Mo bonds in the bimetallic NiMo_2_S_4_ clusters likely provide an additional mode for hydrogen activation and stabilization as sulfhydryl groups on the bridging sulfur atoms. Overall, these IR spectroscopy results suggest that the enhanced ethene hydrogenation activity of NiMoS catalysts is likely due to additional stabilization of H_2_ as sulfhydryl groups on the bridging sulfur atoms of Ni–S–Mo bonds in the bimetallic NiMo_2_S_4_ clusters. This contrasts with the H_2_ activation on monometallic Mo_2_S_4_ clusters, which have been shown to stabilize hydrogen primarily as hydride species on the Mo atoms.^57^

**Figure 8:**
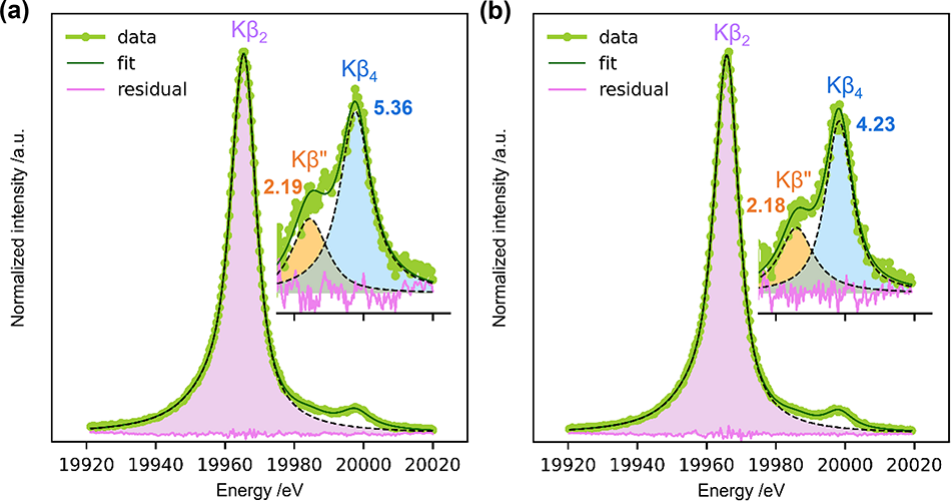
Mo Kβ X-ray emission spectra and Kβ_2_-subtracted spectra (inset) of (a) monometallic MoS(337) (left panel) and (b) bimetallic NiMoS(582,406) (right panel) catalysts. All spectra were measured in situ after sulfidation of the catalyst precursors in 10 vol % H_2_S/H_2_ at 673 K for 2 h. The reported numbers are the relative intensities (in percentage) of Kβ” (orange) and Kβ_4_ (blue) peaks compared to the Kβ_2_ peak (purple).

Figure 8 presents the Mo Kβ X-ray emission spectra of monometallic MoS(337) and bimetallic NiMoS(582,406) catalysts. Prior to the measurements, both catalyst samples were sulfided in 10 vol % H_2_S/H_2_ at 673 K for 2 h. After sulfidation, the spectra were recorded under an inert atmosphere. As expected, all catalyst samples displayed a prominent XES peak at ∼19966 eV, along with two smaller peaks at ∼19988 eV and ∼19998 eV. The main peak at ∼19966 eV corresponds to the Kβ_2_ transitions (i.e., Mo 4*p* → 1*s*).^62,63^ The less intense peaks beyond Kβ_2_ peak are assigned to the Kβ_4_ transitions (i.e., Mo 4*d* → 1*s*) at ∼19998 eV and the valence-to-core Kβ” transitions (i.e., S 3*s* → Mo 1*s*) at ∼19988 eV.^62,63^ The relative intensities of the Kβ” and Kβ_4_ XES peaks, compared to the main Kβ_2_ peak, are also reported in Figure 8.

The relative intensity of the Kβ” peak was nearly identical in both catalyst samples (*I*_*K*β″_/*I*_*K*β_2__ ≈ 2.18–2.19%). However, the relative intensity of the Kβ_4_ peak was significantly lower for the bimetallic NiMoS catalyst (*I*_*K*β_4__/*I*_*K*β_2__ ≈ 4.23%) compared to that for the monometallic MoS catalyst (*I*_*K*β_4__/*I*_*K*β_2__ ≈ 5.36%). We postulate that this decrease in the relative intensity of the Kβ_4_ peak, which corresponds to Mo 4*d* → 1*s* transitions, is likely due to reduced electron density around the Mo atoms in the bimetallic NiMoS clusters.

**Figure 9:**
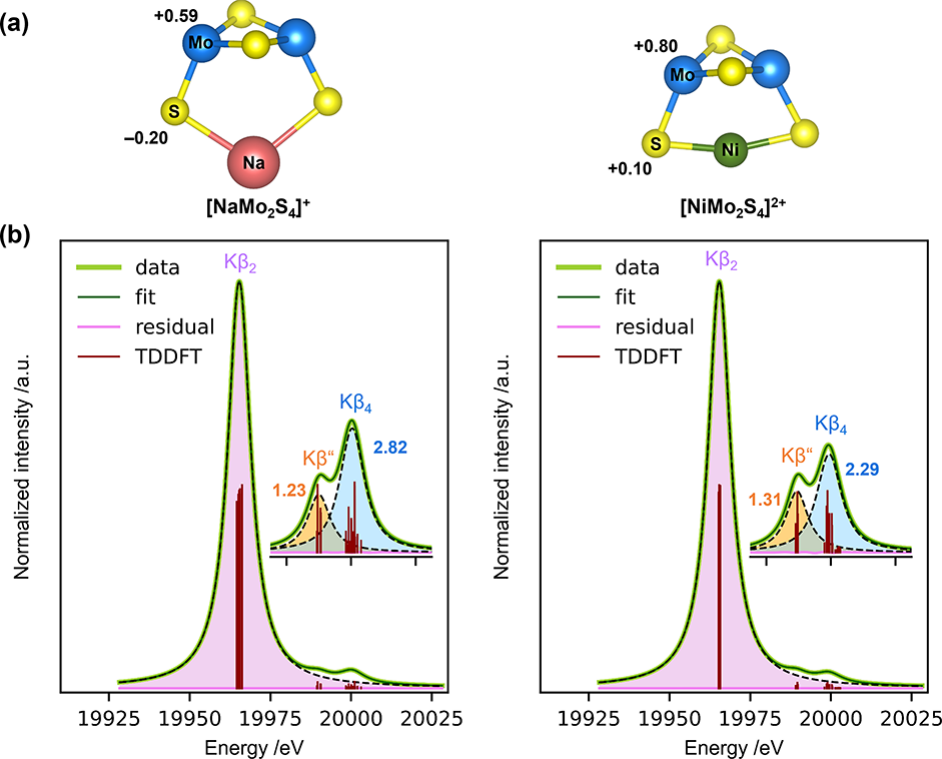
(a) DFT/B3LYP/def2-TZVP-optimized geometries of [NaMo_2_S_4_]^+^ (left) and [NiMo_2_S_4_]^2+^ (right) clusters. The reported numbers are atomic charges estimated from Hirshfeld charge analysis. Mo: blue, S: yellow, Na: red, Ni: green. (b) TDDFT-simulated Mo Kβ X-ray emission spectra and Kβ_2_-subtracted spectra (inset) of [NaMo_2_S_4_]^+^ (left) and [NiMo_2_S_4_]^2+^ (right) clusters. A Lorentzian broadening of 8.5 eV was applied to the X-ray emission lines (red). The simulated spectra were energy-shifted to align the Kβ_2_ feature at 19966 eV. The reported numbers are the relative intensities (in percentage) of the Kβ” (orange) and Kβ_4_ (blue) peaks compared to the Kβ_2_ peak (purple).

To further explore the differences in the electronic and structural properties of the monometallic Mo_2_S_4_ and bimetallic NiMo_2_S_4_ clusters, we employed first-principles DFT simulations. It is important to note here that, similar to the formation of Ni^2+^-coordinated Mo_2_S_4_ clusters in the bimetallic NiMoS catalysts, the dimeric Mo_2_S_4_ clusters in the monometallic MoS catalysts likely interact with the Na^+^ cations present in the faujasite supercage to form [NaMo_2_S_4_]^+^-like species. The DFT-optimized geometries of bare [NiMo_2_S_4_]^2+^ and [NaMo_2_S_4_]^+^ clusters are presented in Figure 9(a).

Figure 9(b) shows the theoretically simulated Mo Kβ X-ray emission spectra of [NaMo_2_S_4_]^+^ and [NiMo_2_S_4_]^2+^ clusters using time-dependent (TD)-DFT. A Lorentzian broadening of 8.5 eV was applied to the theoretical emission lines for comparison with the experimental data. The simulated spectra exhibited characteristic XES features attributable to the Kβ_2_, Kβ_4_ and valence-to-core Kβ” transitions. The simulated spectra were energy-shifted to align the Kβ_2_ feature with the experimentally observed Kβ_2_ peak at ∼19966 eV. The relative intensities of the Kβ” and Kβ_4_ XES peaks were also calculated and are also reported in Figure 9(b). Remarkably, in agreement with the experimental data, the relative intensity of the Kβ” peak was nearly identical in both clusters (*I*_*K*β″_/*I*_*K*β_2__ ≈ 1.23–1.31). However, the relative intensity of the Kβ_4_ peak was lower for [NiMo_2_S_4_]^2+^ cluster (*I*_*K*β_4__/*I*_*K*β_2__ ≈ 2.29) compared to [NaMo_2_S_4_]^+^ cluster (*I*_*K*β_4__/*I*_*K*β_2__ ≈ 2.82). The decrease in the relative intensity of the simulated Kβ_4_ feature, in corroboration with the experimental results, also suggests a lower electron density around the Mo atoms in the bimetallic clusters.

The atomic charges on the Mo and S atoms in the [NaMo_2_S_4_]^+^ and [NiMo_2_S_4_]^2+^ clusters, estimated from Hirshfeld analysis, are also presented in Figure 9(a). We can clearly see that the charges on the Mo and S atoms in [NiMo_2_S_4_]^2+^ cluster were relatively higher compared to those in [NaMo_2_S_4_]^+^. This increase in charge, therefore, indicates that the electron density on the Mo and S atoms in the bimetallic [NiMo_2_S_4_]^2+^ clusters are lower compared to the electron densities in the monometallic [NaMo_2_S_4_]^+^ clusters. This decrease in electron density explains the lower relative intensity of the Kβ_4_ feature in the bimetallic clusters. Finally, we postulate that this lower electron density is likely due to the coordination of the dimeric Mo_2_S_4_ species to Ni^2+^ cations with higher Sanderson electronegativity (*S* = 1.94) compared to that for Na^+^ (*S* = 0.84) or Mo^4+^ (*S* = 1.40).^64^

**Figure 10:**
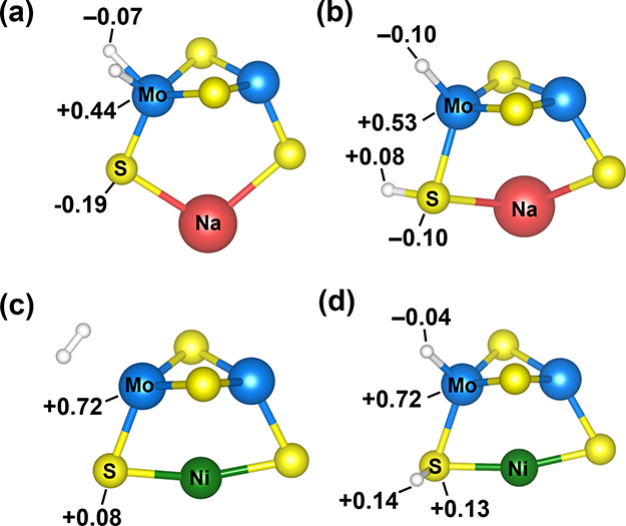
(a,b) DFT/B3LYP/def2-TZVP-optimized geometries of configurations involving dissociatively adsorbed H_2_ on single Mo atom (left) and one Mo atom and one bridging S atom (right) of [NaMo_2_S_4_]^+^ cluster. (c,d) DFT/B3LYP/def2-TZVP-optimized geometries of configurations involving dissociatively adsorbed H_2_ on single Mo atom (left) and one Mo atom and one bridging S atom (right) of [NiMo_2_S_4_]^2+^ cluster. The reported numbers are atomic charges on the specified atoms estimated from Hirshfeld analysis. Mo: blue, S: yellow, Na: red, Ni: green, H: white.

Next, we simulated the dissociative adsorption of H_2_ on the bimetallic [NiMo_2_S_4_]^2+^ and monometallic [NaMo_2_S_4_]^+^ clusters using DFT. For this, we optimized the geometries of [NiMo_2_S_4_]^2+^ and [NaMo_2_S_4_]^+^ clusters together with a H_2_ molecule dissociatively adsorbed on either (i) a single Mo atom or (ii) one Mo and one bridging sulfur atom. The thermodynamically stable configurations are presented in Figure 10. We also simulated the configurations with H_2_ dissociatively adsorbed on one Mo atom and one bridging S atom between the two Mo atoms. The structures and the relevant interatomic distances in all optimized configurations are presented in Supplementary Figures S6 and S7, while the relative standard enthalpies and free energies are reported in Supplementary Tables S5 and S6.

Notably, for the [NaMo_2_S_4_]^+^ cluster, the dissociative adsorption of H_2_ on single Mo atom or one Mo and one bridging sulfur atom was possible, which is consistent with what we reported previously.^57^ However, the configuration with H_2_ dissociatively adsorbed on Mo as hydride species (see Figure 10(a)) was thermodynamically more favorable (see Table S5; Supporting Information). In other words, the formation of sulfhydryl groups (see Figure 10(b)) was thermodynamically less favorable on the Na^+^-coordinated Mo_2_S_4_ clusters.

In contrast, for the bimetallic [NiMo_2_S_4_]^2+^ clusters, the dissociative adsorption of H_2_ on the Mo atoms as hydrides was not thermodynamically feasible. In our simulations, during the geometry optimization, H_2_ could only stabilize as a physisorbed H_2_ molecule near the Mo atom (see Figure 10(c)). On the other hand, the dissociative adsorption of H_2_ on one Mo and one bridging sulfur atom was thermodynamically favorable (see Figure 10(d)). These DFT calculations, therefore, further corroborate that the formation of sulfhydryl groups is favorable on the bimetallic [NiMo_2_S_4_]^2+^ clusters while the monometallic [NaMo_2_S_4_]^+^ clusters primarily stabilize hydrogen as hydrides.

The atomic charges on Mo and S atoms, estimated from Hirshfeld analysis, after H_2_ adsorption are also presented in Figure 10. The formation of hydride species on Mo atoms (Mo^δ+^–H^δ−^) resulted in electron density transfer from the cluster to the H adatoms. On the other hand, formation of sulfhydryl groups (−S^δ−^H^δ+^) resulted in an electron density transfer from H atoms to the cluster. These results suggest that a higher electron density on the TMS clusters would favor the formation of hydrides while a lower electron density would favor the formation of sulfhydryl groups.^57^ Hence, based on the charge transfer analysis, we conclude that stabilization of hydrogen as sulfhydryl groups on the bimetallic NiMo_2_S_4_ clusters is caused by the lower electron density around Mo and S atoms, which in turn is induced by the coordination of Mo_2_S_4_ species to the more electronegative Ni^2+^ cations present in the faujasite supercage.

**Figure 11:**
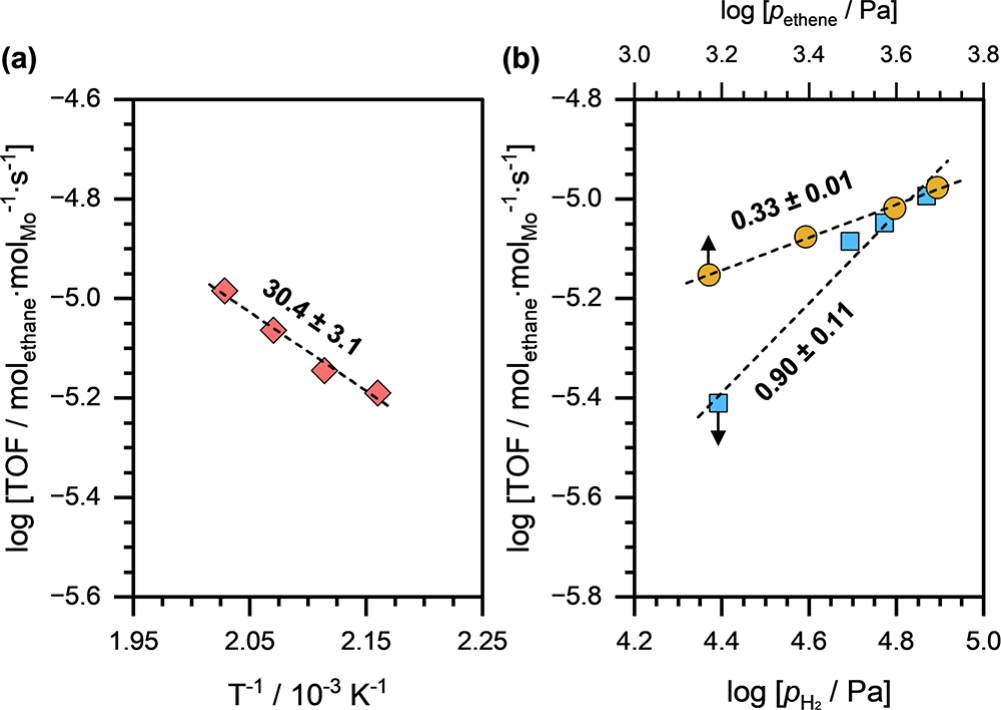
(a) Arrhenius plots (*T* ≈ 463–493 K, *p*_*H*_2__ ≈ 49 kPa, *p*_*ethene*_ ≈ 2.5 kPa) on NiMoS(494,532). The dashed line is a linear fit, and the reported number is the apparent activation energy in kJ·mol^–1^. (b) Steady-state ethane formation rates as a function of H_2_ partial pressure (*T* ≈ 473 K, *p*_*H*_2__ ≈ 25–74 kPa, and *p*_*ethene*_ ≈ 2.5 kPa) and ethene partial pressure (*T* ≈ 473 K, *p*_*H*_2__ ≈ 49 kPa, *p*_*ethene*_ ≈ 1.5–5 kPa) on NiMoS(494,532) catalyst. The dashed lines are linear fits, and the reported numbers are the reaction orders in *p*_*ethene*_ or *p*_*H*_2__.

Finally, we estimated the apparent activation energy for ethene hydrogenation (*E*_*A*, *app*_) and reaction orders in *p*_*ethene*_ and *p*_*H*_2__ for a representative NiMoS(494,532) catalyst and the results are presented in Figure 11. The *E*_*A*, *app*_ and reaction orders for a representative MoS(334) catalyst were reported previously.^57^ The *E*_*A*, *app*_ for the NiMoS catalyst sample was estimated to be approximately 30 kJ·mol^–1^, which is similar to that for the MoS catalyst sample (*E*_*a*, *app*_ ≈ 31 kJ·mol^–1^). The Arrhenius pre-exponential factor (*A*_*app*_) on NiMoS (*A*_*app*_ ≈ 1.6 × 10^–2^ mol_ethane_·mol_Mo_·s^–1^) was, however, significantly higher than that for MoS (*A*_*app*_ ≈ 6.3 × 10^–3^ mol_ethane_·mol_Mo_·s^–1^). Additionally, the NiMoS catalyst displayed slightly lower reaction orders (∼0.90 in *p*_*H*_2__ and ∼0.33 in *p*_*ethene*_) compared to those obtained for the MoS catalyst (∼1.05 in *p*_*H*_2__ and ∼0.56 in *p*_*ethene*_, respectively).

Based on the respective Ni and Mo contents in the representative NiMoS(494,532) catalyst (i.e., ∼494 μmol_Ni_·g_cat_^–1^ and ∼532 μmol_Mo_·g_cat_^–1^, respectively) and assuming a stochastic distribution of isolated Ni and dimeric Mo species within the zeolite micropores, we estimated the concentration of Mo present as NiMo_2_S_4_ clusters to be ∼109 μmol_Mo_·g_cat_^–1^. The remaining Mo, i.e., ∼423 μmol_Mo_·g_cat_^–1^, likely exists as Na^+^-coordinated Mo_2_S_4_ clusters. Assuming identical *E*_*A*, *app*_ and *A*_*app*_ for the Mo_2_S_4_ clusters present in both MoS and NiMoS catalysts, we evaluated their contribution to the net ethene hydrogenation activity. By subtracting this contribution, we estimate the effective *A*_*app*_ on the NiMo_2_S_4_ clusters to be equal to approximately 5.0 × 10^–2^ mol_ethane_·mol_Mo_·s^–1^, almost an order-of-magnitude higher that on the Mo_2_S_4_ clusters, i.e., 6.3 × 10^–3^ mol_ethane_·mol_Mo_·s^–1^.

A significantly higher *A*_*app*_ on NiMo_2_S_4_ clusters compared to Mo_2_S_4_ clusters agrees well with the significantly higher intrinsic TOF on NiMo_2_S_4_ clusters (∼68 mol_ethane_·mol_cluster_^–1^·h^–1^) than that on Mo_2_S_4_ (∼6.7 mol_ethane_·mol_cluster_^–1^·h^–1^). The Arrhenius pre-exponential factor can be regarded as a qualitative indicator of the frequency of reactive collisions between the reactants on the active sites. Hence, the significantly higher *A*_*app*_ on NiMo_2_S_4_ clusters suggests higher availability of adsorbed hydrogen on these clusters, likely due to its different mode of stabilization on the bridging sulfur atoms as sulfhydryl groups. We postulate that the hydrogen coverage on monometallic Mo_2_S_4_ clusters as hydrides on the Mo atoms is relatively low due to its competition with ethene for the same sites. On the other hand, the stabilization of hydrogen as sulfhydryl groups on the bridging sulfur atoms likely does not compete with ethene for the same sites and thus results in higher coverage of adsorbed hydrogen.

## Conclusions

Bimetallic Ni and Mo-based TMS clusters encapsulated within the micropores of a faujasite-type Y zeolite have been synthesized by first partly ion exchanging the NaY zeolite with Ni^2+^ cations, followed by CVD of Mo(CO)_6_ and subsequent sulfidation in H_2_S/H_2_ at 673 K. The sulfidation resulted in the formation of homogeneously distributed dimeric Mo_2_S_4_ clusters. Additionally, Ni exists in two distinct forms: (i) as sulfur-coordinated isolated Ni^2+^ species homogeneously distributed within the zeolite micropores and (ii) as small bulk Ni sulfide nanoparticles predominantly on the external surface of the zeolite crystallite. The presence of isolated Ni^2+^ cations and dimeric Mo sulfide species in the same faujasite supercage, during the sulfidation treatment, resulted in the formation of bimetallic NiMo_2_S_4_-like clusters with bridging sulfur atoms connecting Ni and Mo.

The bimetallic NiMo_2_S_4_ clusters exhibit a different mode of H_2_ adsorption and stabilization compared to monometallic Mo_2_S_4_ clusters. While the latter stabilized adsorbed hydrogen primarily as hydrides on the Mo atoms, the bimetallic NiMo_2_S_4_ clusters stabilized hydrogen as sulfhydryl groups on the sulfur atoms bridging between two TMs. The stabilization of hydrogen as sulfhydryl groups is enabled by the lower electron density on the cluster induced by the coordination of the dimeric Mo_2_S_4_ cluster to more electronegative Ni^2+^ cations. The overall rates of ethene hydrogenation were significantly higher on the bimetallic TMS catalysts due to the different mode of hydrogen adsorption that does not compete with ethene for the same sites, resulting in higher availability of adsorbed hydrogen.

## Methods

### Catalyst Precursor Preparation

NaY zeolite (Zeolyst CBV 100, Si/Al ∼2.4) was ion-exchanged with Ni^2+^ cations. For this, approximately 1 g of NaY zeolite was suspended in ∼50 mL of 10–20 mM nickel(II) acetate solution. The suspension was stirred at 353 K overnight and the solid was filtered and collected, subsequently washed with excess water (3 × 50 mL), and dried at 373 K overnight. The ion-exchanged zeolites (referred to NiNaY(X) where “X” refers to the Ni content, determined from elemental analysis, expressed in μmol_Ni_·g_cat_^–1^) were finally calcined in synthetic air (20 vol % O_2_/N_2_) at 823 K (temperature ramp ramp: 5 K·min^–1^ to 823 K) for 10 h.

The NiNaY zeolites were then impregnated with Mo(CO)_6_ (≥99.9%; Sigma-Aldrich) via chemical vapor deposition (CVD). Briefly, approximately 200 mg zeolite (pelletized and sieved between 250–355 μm) were treated under reduced pressure (<10^–2^ mbar) at elevated temperatures (temperature ramp: 5 K·min^–1^ to 408 K, hold for 2 h; 5 K·min^–1^ to 503 K, hold for 2 h; 5 K·min^–1^ to 653 K, hold for 1 h) to carefully remove any adsorbed water. Mo(CO)_6_ was then loaded on the dried zeolite at room temperature under static conditions for a predefined time period. In a final step, the catalyst precursors were evacuated under reduced pressure (<10^–2^ mbar) for ∼10 min to remove physisorbed Mo(CO)_6_ species. All catalyst precursors were stored in a glovebox to avoid exposure to air/moisture.

### Catalytic Reactions

All reactions were carried out in a lab-scale plug flow reactor (PFR) constructed out of quartz tube (ϕ_*i*.*d*._ ≈ 4 mm). Bronkhorst electronic mass flow controllers (MFCs) were used to control the flow rates of gases. All catalysts were diluted 1:10 in silicon carbide (sieved between 500–1000 μm) and placed in the reactor tube supported between two quartz wool plugs. The catalyst precursors were first sulfided in 20 mL·min^–1^ 10 vol % H_2_S/H_2_ at ambient pressure and 673 K (temperature ramp: 5 K·min^–1^ to 673 K) for 2 h. After sulfidation, all catalysts were purged with N_2_ for 30 min prior to catalytic reactions. Ethene hydrogenation was performed at ∼473 K and ambient pressure in the absence of H_2_S in the feed with a H_2_/C_2_H_4_ volumetric ratio of ∼19. Composition of the product stream was analyzed by an online Agilent 7890B Gas Chromatograph (GC). Ethane was detected as the only product. Refer to Supporting Information**Section S1** for additional details.

### Infrared Spectroscopy of Adsorbed Probe Molecules

Infrared (IR) spectroscopy of adsorbed probe molecules was performed on a Nicolet 6700 FT-IR spectrometer with a resolution of 4 cm^–1^. Prior to the IR experiments, all catalyst precursors were sulfided in the PFR setup. After sulfidation, catalysts were ground and pressed into self-supporting wafers (ρ_*A*_ ≈ 5 mg·cm^–2^). Prior to measurements, the wafers were resulfided in a stream of 20 mL·min^–1^ 10 vol % H_2_S/H_2_ at ambient pressure and 673 K (temperature ramp: 5 K min^–1^ to 673 K) for 2 h. Finally, the catalyst wafers were activated in H_2_ (4 cycles, total 24 h) at 473 K each followed by evacuation at 10^–6^ mbar for at least 30 min.

Pyridine was adsorbed on the activated samples at 323 K by applying small doses (*p*_*Py*_ ≈ 0.01–0.5 mbar) into the IR cell followed by equilibration for at least 30 min. After adsorption, the samples were outgassed at 323 K and 10^–6^ mbar for 30 min. Pyridine was desorbed by heating in steps of 50 K and equilibrating for 30 min after each step.

Similarly, 2,4-dimethylpyridine (DMP) was adsorbed on the activated samples at 323 K by applying small doses (up to *p*_*DMP*_ ≈ 1 mbar). After adsorption, the samples were outgassed at 323 K and 10^–6^ mbar for 30 min. A second spectrum was collected after applying an additional 1 bar of H_2_, followed by equilibration for 30 min and outgassing at 10^–6^ mbar for 30 min.

The IR spectra were background corrected using OMNIC software package. All spectra are reported as the difference spectra against a reference spectrum on a clean zeolite measured at 10^–7^ mbar.

### X-ray Absorption Spectroscopy

X-ray absorption spectroscopy (XAS) measurements were performed at the P65 beamline of the German Electron Synchrotron (DESY) in Hamburg, Germany.^65^ The storage ring operated at 6 GeV energy and 100 mA current in top-up mode. A water-cooled Si111 double crystal monochromator (DCM) was used for obtaining monochromatic X-rays. Two Si or Rh-coated plane mirrors were installed in front of the DCM to reject the higher harmonics. The DCM was calibrated by measuring the respective metal foils and setting the first major inflection point to the theoretical edge energy (20000 eV for Mo and 8333 eV for Ni). The beam spot-size was 300 μm (vertical) × 1.5 mm (horizontal) on the sample. Refer to Supporting Information Section S1 for additional details.

### X-ray Emission Spectroscopy

X-ray emission spectroscopy (XES) measurements were performed at the ID26 beamline of the European Synchrotron Radiation Facility (ESRF) in Grenoble, France. The storage ring operated at 6 GeV energy and 90 mA current. A flat Si311 DCM was used for obtaining monochromatic X-rays. A water cooled Rh-coated plane mirror was used to reject higher harmonics. The beam spot-size was approximately 100 μm (vertical) × 100 μm (horizontal) at the sample position. XES data were collected using a dead-time corrected silicon drift diode detector (from Ketek). Possible attenuation in emission signal was minimized by placing a He-filled balloon inside the Rowland circle between the sample, the analyzer crystals, and the detector. Refer to Supporting Information Section S1 for additional details.

### Computational Methods

Density functional theory (DFT) calculations were performed with the hybrid exchange-correlational functional B3LYP using the Orca quantum chemistry package version 5.0.3.^66^ Refer to Supporting Information Section S1 for additional computational details.

## Supporting Information Available

Additional experimental and computational details, additional figure and tables, cartesian coordinates of optimized structures, example Orca input files (PDF)

## ABBREVIATIONS

TMS: transition metal sulfides
CVD: chemical vapor deposition
TOF: turnover frequency
DFT: density functional theory
XAS: X-ray absorption spectroscopy
XES: X-ray emission spectroscopy
XANES: X-ray absorption near edge structure
EXAFS: extended X-ray absorption fine structure
TOS: time-on-stream
EDXS: energy dispersive X-ray spectroscopy
HDS: Hydrodesulfurization
CUS: coordinatively unsaturated sites
BAS: Bro̷nsted acid sites
LAS: Lewis acid sites
HAADF-TEM: high-angle annular dark field-transmission electron microscopy
CN: coordination number
DMP: 2,4-dimethylpyridine
IR: infrared
