# Supplementary material for: Hydrogen Activation on Zeolite Stabilized Ni–Mo Sulfide Clusters
Source: JACS Au. 2025 Jan 24;5(2):890–901. doi: 10.1021/jacsau.4c01088 (PMC11862920; doi:10.1021/jacsau.4c01088)
Supplement: Supplementary file 1 — au4c01088_si_001.pdf [file au4c01088_si_001.pdf]

Supporting Information for

Hydrogen Activation on Zeolite Stabilized Ni–Mo Sulfide Clusters

Rachit Khare,*[,a,†] Roland Weindl,[a,†] Sungmin Kim,[b] Libor Kovarik,[b] Andreas Jentys,[a] Karsten Reuter[c] and Johannes A. Lercher*[,a,b]

[a]Department of Chemistry and Catalysis Research Center, Technical University of Munich, 85748 Garching, Germany.

[b]Institute for Integrated Catalysis, Pacific Northwest National Laboratory, Richland, WA 99352, United States.

[c]Theory Department, Fritz Haber Institute of the Max Plank Society, 14195 Berlin, Germany.

[†]These authors contributed equally to the work.

*Corresponding authors: rachit.khare@tum.de, johannes.lercher@pnnl.gov, johannes.lercher@ch.tum.de

Table of Contents

S1. Additional Experimental and Computational Details 3

S2. Additional Tables and Figures 6

S3. Additional Simulation Results 15

S4. Cartesian Coordinates of the DFT-Optimized Structures 19

S5. Example Orca Input Files.. 23

References.. 25

S1. Additional Experimental and Computational Details

S1.1. Activation energy measurement.

The apparent activation energy ($E_{a,app}$) and Arrhenius pre-exponential factors (A_{app}) were determined from the Arrhenius-type plots ($\log r$ *versus* T^{-1}), using the following equations:

$$E_{a,app} = 2.303 \cdot m \cdot R$$

$$A_{app} = c$$

where m and c are the slope and the y-intercept of the linear regression fit to the Arrhenius plots and R is the universal gas constant (= 8.314 $J \cdot mol^{-1} \cdot K^{-1}$).

S1.2. High-angle annular dark field-transmission electron microscopy & energy dispersive X-ray spectroscopy.

Transmission electron microscopy (TEM) sample preparation involved crushing the catalyst powder in dry form between two glass slides and subsequently dispersing the powder directly onto Cu 300 mesh lacey carbon TEM grids. The as-prepared TEM grids were then transferred to a TEM holder and loaded into the microscope for imaging. S/TEM analysis was performed with aberration corrected Thermo-Fisher Themis Z Scanning/Transmission Electron Microscope (S/TEM) operated at 300 kV. The probe convergence angle was 25 mrad, and the inner detection angle on the HAADF detector of 52 mrad. Compositional analysis was performed with Thermo-Fischer Super-X silicon drift detector. The detector consists of 4 quadrants with a total of ~0.8 Steradian collection angle. The acquisition and processing were performed with Thermo-Fisher's Velox software.

S1.3. X-ray absorption spectroscopy measurements.

X-ray absorption spectra were measured using a quartz capillary micro-reactor setup. Prior to the measurements, catalyst precursors were placed in a quartz capillary (WJM Glas, $\phi_{o.d.} \approx 1$ mm, 10–20 μm thickness) supported between two quartz wool plugs. The capillary was heated from below with a hot-air gas-blower (Oxford FMB). Gas flow rates were controlled using Bronkhorst MFCs. All experiments were performed under ambient

pressure. The precursors were sulfided in situ under 10 mL·min^{-1} 10 vol.% H_2S/ H_2 flow at 673 K (temperature ramp: 5 K·min^{-1} to 673 K) for 2 h. After sulfidation, the capillary was cooled down to room temperature and placed on the sample stage for XAS measurements, while maintaining the gas flow.

The data were monitored for any signs of X-ray beam damage and several successive scans were averaged to improve the data quality. The spectra were measures in both total fluorescence yield (TFY) mode using a passivated implanted planar silicon (PIPS) detector and in transmission mode using gas ionization chambers.

For X-ray absorption near-edge structure (XANES) analysis, the spectra were normalized and flattened. For extended X-ray absorption fine structure (EXAFS) analysis, spectra were background subtracted, normalized, k^2-weighted, and Fourier-transformed (FT) using the Athena software package.[1] The EXAFS fitting was performed in k-space simultaneously on the k^1-, k^2-, and k^3-weighted data, using the Artemis software package.[1] The reference metal foils were first fitted to their theoretical structures to obtain the corresponding amplitude reduction factors, S_0^2, which were then used in the subsequent fits.

S1.4. X-ray emission spectroscopy measurements.

X-ray emission spectra were measured using a similar quartz capillary micro-reactor setup as described above for XAS measurements. The catalyst precursors were placed in a quartz capillary and sulfided in 10 mL min^{-1} 10 vol.% H_2S/H_2 at 673 K (temperature ramp: 5 K·min^{-1} to 673 K) for 2 h. After sulfidation, the capillary was placed on the sample stage and cooled down using a liquid N_2 cryo-stream. The X-ray emission spectra were collected on the sulfided samples under an inert atmosphere. The data were monitored for any signs of X-ray beam damage and several successive scans were averaged to improve the data quality.

S1.5. Computational details.

Density functional theory (DFT) calculations were performed with the hybrid exchange-correlational functional B3LYP using the Orca quantum chemistry package version

5.0.3.[2] Grimme's atom-pairwise dispersion corrections with the Becke-Johnson damping scheme (D3BJ) were used in all calculations.[3, 4] RIJCOSX approximation was employed to speed up the calculations and auxiliary basis sets def2/J or SARC/J were used for this purpose.[5, 6] The RIJCOSX approach incorporates the resolution-of-identity (RI) approximation for the evaluation of the Coulomb matrices and the chain-of-spheres algorithm for the formation of the exchange-type matrices.[7]

Weigend and Ahlrichs' def2-TZVP basis sets were used for geometry optimization,[8] while relativistically recontracted versions of the all-electron Ahlrichs def2 basis sets with triple zeta polarization functions (ZORA-def2-TZVPP) were employed for other calculations. The electron density-based Hirshfeld charge and spin population analysis was performed on the DFT-optimized geometries. The X-ray emission spectra of the optimized structures were simulated with time-dependent (TD)-DFT using the Tamm-Dancoff approximation. The calculated intensities included the electric dipole, the magnetic dipole and the electric quadrupole contributions. A Lorentzian broadening of 8.5 eV was applied to the theoretical X-ray emission lines for comparison with the experimental data. Example Orca input files used for geometry optimization, single-point energy calculations, and XES simulations are provided in **Section S5**.

S2. Additional Tables and Figures

Table S1. Ni and Mo content, expressed in $\mu mol_{metal} \cdot g_{cat}^{-1}$, in different catalyst samples investigated in this work.

Catalyst	Parent zeolite	Metal content /$\mu mol_{metal} \cdot g_{cat}^{-1}$	
		**Mo**	**Ni**
NiS(665)	NiNaY(665)	0	665
NiS(51)	NiNaY(51)	0	51
NiMoS(612,164)	NiNaY(665)	164	612
NiMoS(583,406)	NiNaY(665)	406	583
NiMoS(540,511)	NiNaY(665)	511	540
NiMoS(494,532)	NiNaY(665)	532	494
NiMoS(51,524)	NiNaY(51)	524	51
NiMoS(172,319)	NiNaY(239)	319	172

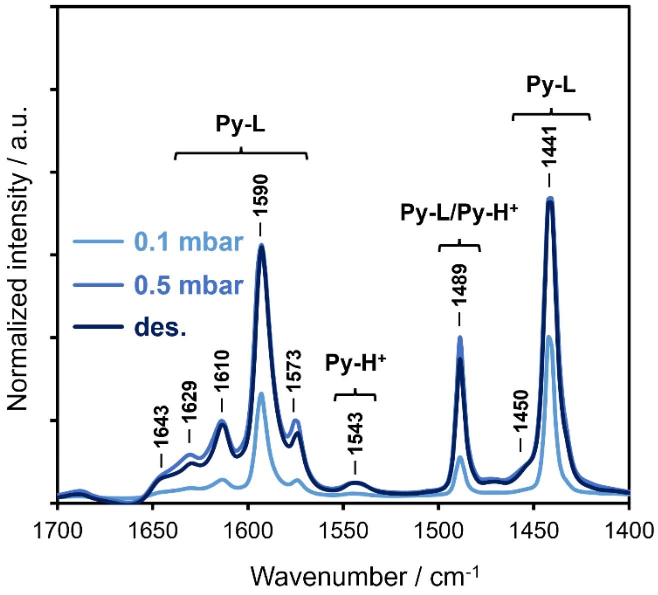

Figure S1. Infrared (IR) spectra of pyridine adsorbed on NiS(665) zeolite sample. All spectra are normalized to catalyst wafer thickness and mass. The catalyst was sulfided in situ prior to the measurements in 10 vol.% H_2S/H_2 at 623 K.

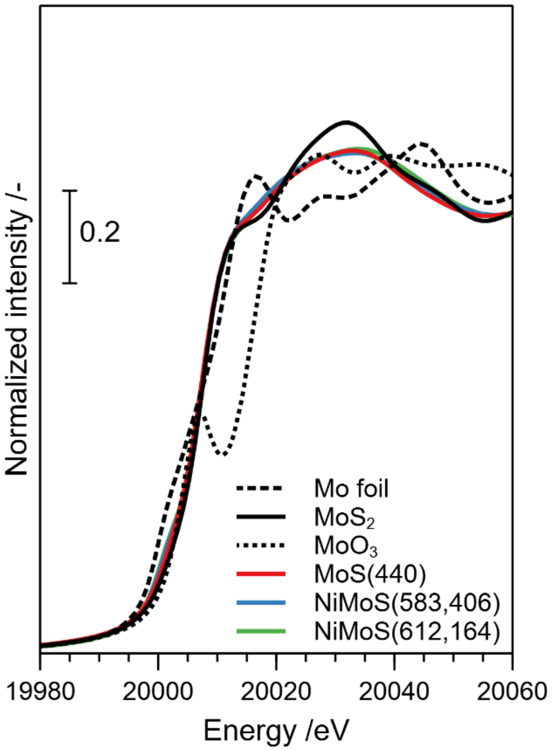

Figure S2. Mo K-edge XANES of MoS(440), NiMoS(583,406) and NiMoS(612,164) catalyst samples. All spectra were measured in situ following the sulfidation of the catalyst precursors in 10 vol.% H_2S/H_2 at 673 K for 2 h. The Mo K-edge XANES of commercially available Mo foil, MoS_2 and MoO_3 reference materials are also shown.

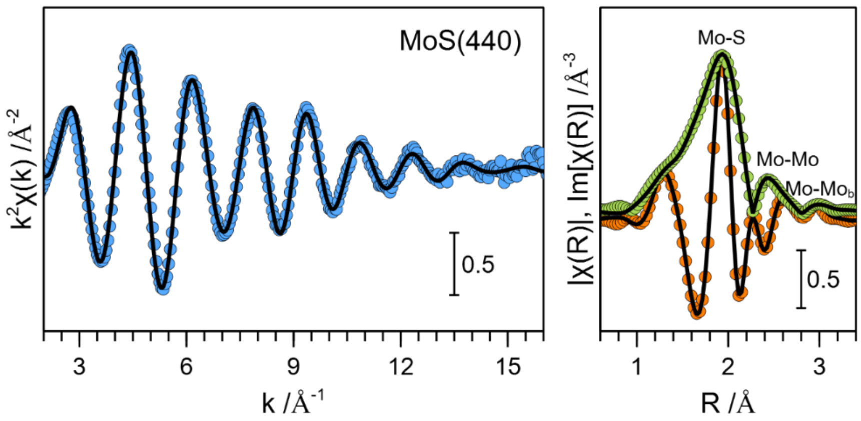

Figure S3. Mo K-edge k^2-weighted EXAFS (left panel) and FT-EXAFS (right panel) of MoS(440) catalyst sample. The spectra were measured in situ following the sulfidation of the catalyst precursor in 10 vol.% H_2S/H_2 at 673 K for 2 h.

Table S2. Mo K-edge EXAFS fitting parameters: coordination numbers (CN), interatomic distances (d), and the Debye-Waller factors (σ^2), for MoS(440), NiMoS(583,406) and NiMoS(612,164) catalysts. Additional fitting parameters: k-range: 2.7–15 Å^{-1}, R-range: 1.0–3.5 Å, $S_0^2 = 1.03$.

Sample	Path	CN	d /Å	ΔE_0 /eV	σ^2 /Å^2
MoS(440)	Mo–S	3.82 ± 0.33	2.423 ± 0.01	2.1 ± 1.0	0.0064 ± 0.0007
	Mo–Mo	0.95 ± 0.25	2.767 ± 0.01		0.0089*
	Mo–Mo_b	0.25 ± 0.12	3.175 ± 0.03		0.006*
	Mo–O	0.29 ± 0.19	1.605 ± 0.07		0.006*
NiMoS(583,406)	Mo–S	3.82 ± 0.34	2.382 ± 0.01	0.1 ± 1.1	0.0075 ± 0.0007
	Mo–Mo	0.87 ± 0.15	2.795 ± 0.01		0.0063*
	Mo–Ni	0.39 ± 0.22	3.286 ± 0.04		0.006*
	Mo–O	0.32 ± 0.13	1.603 ± 0.03		0.006*
NiMoS(612,164)	Mo–S	4.22 ± 0.51	2.376 ± 0.01	0.6 ± 1.4	0.0087 ± 0.0011
	Mo–Mo	1.09 ± 0.19	2.787 ± 0.01		0.0063*
	Mo–Ni	0.23 ± 0.28	3.295 ± 0.07		0.006*
	Mo–O	0.33 ± 0.16	1.599 ± 0.03		0.006*

*This parameter was constrained during the fit.

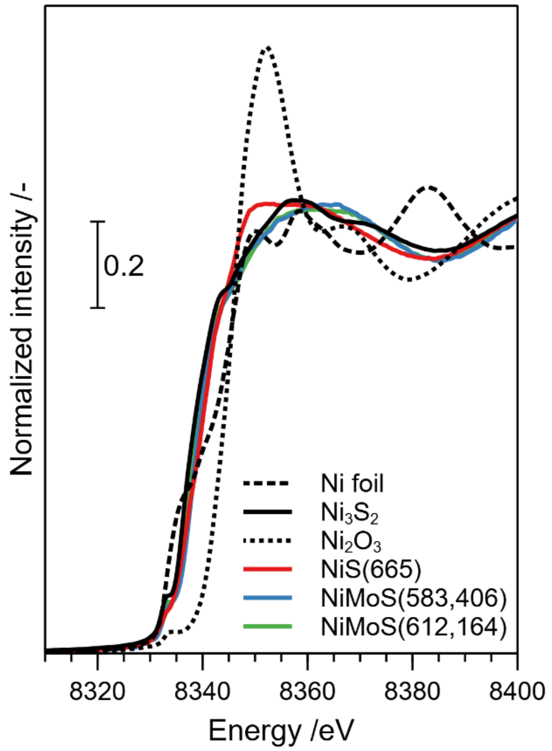

Figure S4. Ni K-edge XANES of NiS(665), NiMoS(583,406) and NiMoS(612,164) catalyst samples. All spectra were measured in situ following the sulfidation of the catalyst precursors in 10 vol.% H_2S/H_2 at 673 K for 2 h. The Mo K-edge XANES of commercially available Ni foil, Ni_3S_2 and Ni_2O_3 reference materials are also shown.

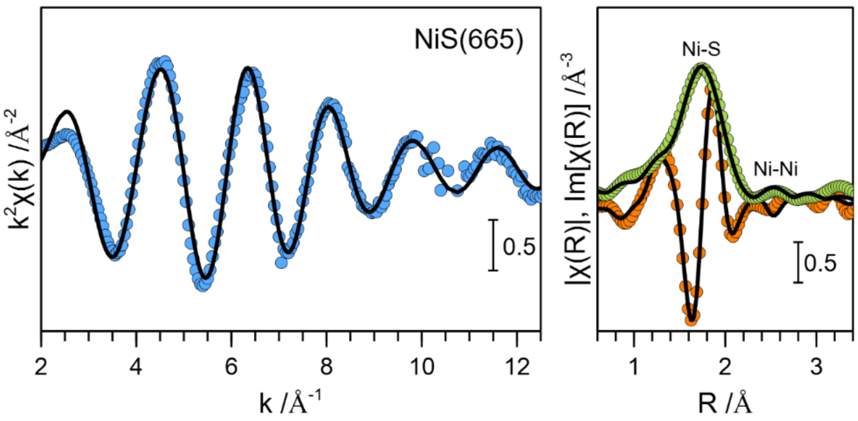

Figure S5. Ni K-edge k^2-weighted EXAFS (left panel) and FT-EXAFS (right panel) of NiS(665) catalyst samples. The spectra were measured in situ following the sulfidation of the catalyst precursors in 10 vol.% H_2S/H_2 at 673 K for 2 h.

Table S3. Ni K-edge EXAFS fitting parameters: coordination numbers (CN), interatomic distances (d), and the Debye-Waller factors (σ^2) for NiMoS catalysts with varying Ni/Mo ratios. Additional fitting parameters: k-range: 2.7–15 Å^{-1}, R-range: 1.0–3.5 Å, $S_0^2 = 0.70$.

Sample	Path	CN	d / Å	ΔE_0 / eV	σ^2 / Å²
NiS(665)	Ni–S	3.56 ± 0.35	2.184 ± 0.009	–3.1 ±1.1	0.0040 ± 0.0011
	Ni–Ni	0.98 ± 0.32	2.499 ± 0.022		0.006*
NiMoS(583,406)	Ni–S	3.94 ± 0.43	2.232 ± 0.009	1.3 ± 1.5	0.0056 ± 0.0008
	Ni–Ni	1.14 ± 0.52	2.538 ± 0.011		0.0046 ± 0.0024
	Ni–S_2	0.27 ± 0.58	3.526 ± 0.141		0.006*
NiMoS(612,164)	Ni–S	4.19 ± 0.33	2.232 ± 0.007	1.0 ± 1.1	0.0063 ± 0.0006
	Ni–Ni	1.15 ± 0.43	2.525 ± 0.009		0.0054 ± 0.0020
	Ni–S_2	0.34 ± 0.43	3.503 ± 0.081		0.006*

*This parameter was constrained during the fit.

Table S4. Mo_2S_4 and Ni content, expressed in atoms/clusters per supercage, in different NiMoS catalyst with varying Mo loadings. The stochastically predicted likelihood of Ni and Mo_2S_4 clusters to exist in the same supercage is also provided.

Catalyst	Mo_2S_4 content /clusters·supercage^{-1}	Ni content /atoms·supercage^{-1}	Stochastic probability
NiMoS(612,164)	0.13	0.49	0.063
NiMoS(583,406)	0.32	0.46	0.150
NiMoS(540,511)	0.41	0.43	0.175
NiMoS(494,532)	0.42	0.39	0.166
NiMoS(51,524)	0.42	0.04	0.017

S3. Additional Simulation Results

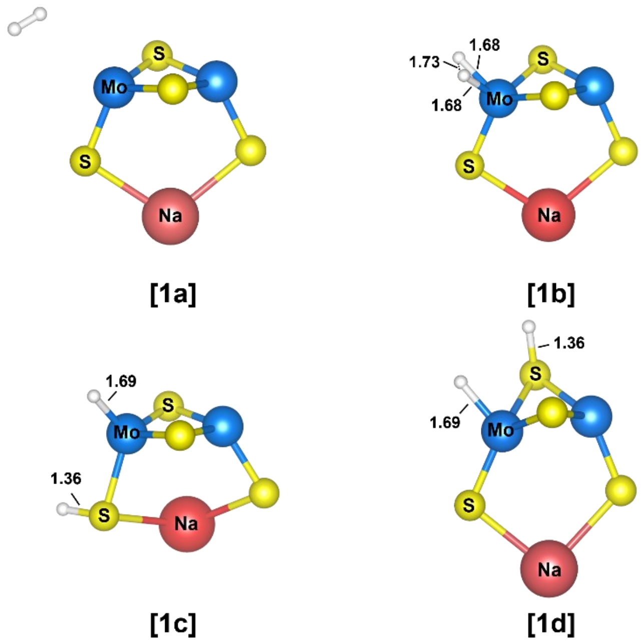

Figure S6. DFT/B3LYP/def2-TZVP-optimized geometries of $[NaMo_2S_4]^+$ clusters with gas-phase H_2 (**[1a]**) or with dissociatively adsorbed H_2 on (i) a single Mo atom (**[1b]**), (ii) one Mo atom and one S atom (**[1c]**), and (iii) one Mo atom and one bridging S atom (**[1d]**). The reported numbers are the interatomic distances in Å. S: yellow; Mo: blue; H: white, Na: red.

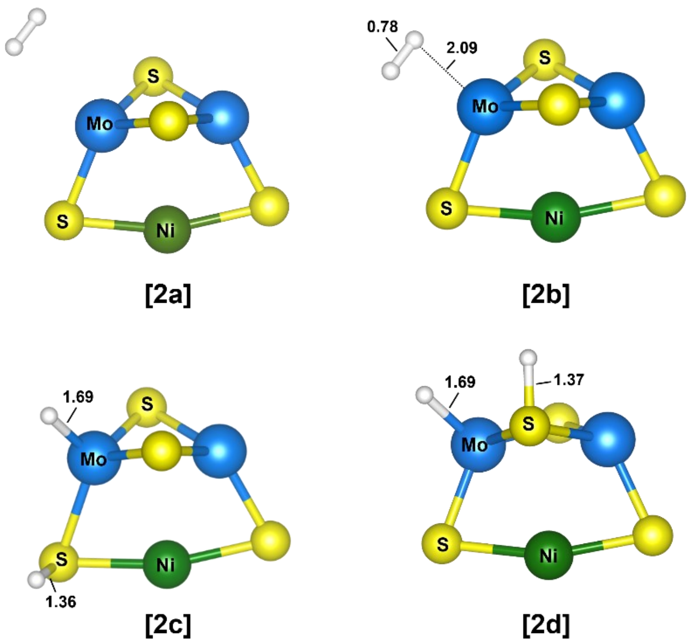

Figure S7. DFT/B3LYP/def2-TZVP-optimized geometries of $[NiMo_2S_4]^{2+}$ clusters with gas-phase H_2 (**[2a]**) or with dissociatively adsorbed H_2 on (i) a single Mo atom (**[2b]**), (ii) one Mo atom and one S atom (**[2c]**), and (iii) one Mo atom and one bridging S atom (**[2d]**). The reported numbers are the interatomic distances in Å. S: yellow; Mo: blue; H: pink, Ni: green.

Table S5. Relative standard enthalpies ($\Delta\Delta H^0$) and standard free energies ($\Delta\Delta G^0$) of the $[NaMo_2S_4]^+$ clusters with gas-phase or dissociatively adsorbed H_2 at different locations, computed at $T = 298$ K and $p_{H_2} = 1$ bar with DFT/B3LYP/def2-TZVPP. The corresponding structures are depicted in **Figures S6**.

Structure	$\Delta\Delta H^0$ /kJ·mol^{-1}	$\Delta\Delta G^0$ /kJ·mol^{-1}
**[1a]**	0.0[a]	0.0[a]
**[1b]**	–67.2	–31.4
**[1c]**	–24.3	9.1
**[1d]**	–7.5	25.4

[a]Enthalpy and free energy of bare cluster with gas-phase H_2 (**[1a]**) was used as the standard and was set to zero.

Table S6. Relative standard enthalpies ($\Delta\Delta H^0$) and standard free energies ($\Delta\Delta G^0$) of $[NiMo_2S_4]^{2+}$ clusters with gas-phase or dissociatively adsorbed H_2 at different locations, computed at $T = 298$ K and $p_{H_2} = 1$ bar with DFT/B3LYP/def2-TZVPP. The corresponding structures are depicted in **Figures S7**.

**Structure**	$\Delta\Delta H^0$ **/kJ·mol⁻¹**	$\Delta\Delta G^0$ **/kJ·mol⁻¹**
**[2a]**	0.0[a]	0.0[a]
**[2b]**	-[b]	-[b]
**[2c]**	–35.3	–0.5
**[2d]**	–16.7	19.3

[a]Enthalpy and free energy of bare cluster with gas-phase H_2 (**[2a]**) was used as the standard and was set to zero.
[b]The structure was not stable.

S4. Cartesian Coordinates of the DFT-Optimized Structures

S4.1. Cartesian coordinates of structure **[1a]**. The DFT/B3LYP/def2-TZVP-optimized geometry is presented in **Figure S6**.

Mo	0.92740049430158	1.88800157681600	-0.66018508123134
Mo	-1.12721507204460	0.28790246242341	-1.50901257377659
S	-1.11123134696511	1.66461135500708	0.27640059254020
S	1.06267326839312	0.06280479387717	-1.97580196873742
Na	-0.72977953442924	3.30614483968189	-3.78951168064371
S	-2.19838278176875	1.10188829281935	-3.16276047435483
S	1.11633497251300	3.69682667937508	-1.77031881379630

S4.2. Cartesian coordinates of structure **[1b]**. The DFT/B3LYP/def2-TZVP-optimized geometry is presented in **Figure S6**.

Mo	0.80759631812006	1.94088953388198	-0.82053535651332
Mo	-1.21192736786572	0.25588616549153	-1.57215888508706
S	-1.25335017838990	1.55107977990389	0.23690381445154
S	0.93411263009636	-0.02999877601905	-2.07768602231458
Na	-0.93169802649637	3.62440693383303	-3.74082198423682
S	-2.21432549594418	1.18848528722677	-3.19844433065585
S	1.05496770191070	3.77004741640309	-1.87756649124914
H	1.28678050853489	2.21788582141251	0.76188991442453
H	2.33676391003415	1.43042783786626	-0.36353065881928

S4.3. Cartesian coordinates of structure **[1c]**. The DFT/B3LYP/def2-TZVP-optimized geometry is presented in **Figure S6**.

Mo	0.69118501984655	1.95563069199031	-1.10283405556331
Mo	-0.87987183786629	0.34887417442229	-1.93229912107439
S	-1.22084856831985	1.47915766228969	0.06047417281063
S	1.42890061691438	-0.25720937629440	-1.94488060746327
Na	-0.99480191909003	4.03424672948251	-3.90098220834045
S	-1.83426630290212	1.37892298099056	-3.53975494214044
S	0.96608837467173	3.86016338870863	-2.06949703360332
H	1.91211870117696	1.48709875595744	-0.02243949230576
H	1.64885591556867	-1.02727500754704	-0.84686671231968

S4.4. Cartesian coordinates of structure **[1d]**. The DFT/B3LYP/def2-TZVP-optimized geometry is presented in **Figure S6**.

Mo	0.88899673738922	1.91096970031563	-0.70718167292787
Mo	-1.00692079742421	0.48600544169577	-1.75949574457070
S	-1.02173694816503	1.21360656688363	0.33991702498761
S	1.12516991192907	0.76188739587787	-2.63755017365692
Na	-0.58364322495207	3.30696077542773	-3.63862187405911
S	-2.55266369200560	1.52393808940030	-2.80635243922179
S	0.49614428903751	4.15182883984687	-1.11474470372695
H	1.60192608970956	1.43370211423539	0.75384571450073
H	1.77369763448155	4.54762107631673	-1.34171613132495

S4.5. Cartesian coordinates of structure **[2a]**. The DFT/B3LYP/def2-TZVP-optimized geometry is presented in **Figure S7**.

Mo	0.84246069434752	1.28290957405337	-1.36828349303196
Mo	-1.37606341219440	0.23511539746354	-1.85457867396651
S	-1.14458943277539	2.18036788909115	-0.65902269971535
S	0.56116212159374	-0.97509623456208	-1.64152967220517
Ni	-0.15733185341699	1.53409628452017	-3.78833658262924
S	-2.05151242100993	0.50728155911923	-3.87552197718397
S	1.73259430345545	2.27673553031462	-3.05095690126780

S4.6. Cartesian coordinates of structure **[2b]**. The DFT/B3LYP/def2-TZVP-optimized geometry is presented in **Figure S7**.

Mo	0.62875099216267	1.68168630038370	-1.27240145171221
Mo	-1.48738800741147	0.36583941513807	-1.86771129907688
S	-1.50059893485844	2.50463790561234	-1.06246273157206
S	0.45833420043640	-0.63191119043159	-1.25333392203989
Ni	-0.01685765498315	1.30155652202006	-3.81838774755531
S	-1.89752233427245	0.24357357101686	-3.97116478483449
S	1.60959046257595	2.46906495069534	-3.01128715155390
H	2.15136568549844	1.08421451152689	0.02332749947008
H	2.51177559085205	1.65779801403831	-0.36262841112528

S4.7. Cartesian coordinates of structure **[2c]**. The DFT/B3LYP/def2-TZVP-optimized geometry is presented in **Figure S7**.

Mo	1.16477692446472	1.20365234874149	-0.56960271428742
Mo	-1.19123946076868	0.54660397131724	-1.55236873928664
S	-0.83533907024352	2.48910614171647	-0.16529516229278
S	0.48184958450377	-0.91894087595946	-1.32076208944870
Ni	0.37286472597286	2.09771612677383	-2.98453611714458
S	-1.43271066301443	1.13487125607443	-3.59391834007255
S	2.28587776155643	2.39251720905640	-1.92954426170623
H	1.38813964051263	1.83261174339881	0.97957140461711
H	-1.19275944298378	1.91479207888078	1.02023601962181

S4.8. Cartesian coordinates of structure **[2d]**. The DFT/B3LYP/def2-TZVP-optimized geometry is presented in **Figure S7**.

Mo	0.64770632891567	-0.21699554203979	0.82135282485878
Mo	-1.55430859964425	-1.16999068686696	-0.08911955978807
S	-1.43051118966257	0.52340206136098	1.40519165304186
S	0.40674241453153	-2.37969981930282	0.28881288051450
Ni	-0.24792173668464	0.44544591810506	-1.60395652811942
S	-2.10722833407167	-0.57214842795330	-2.07544890029351
S	1.78685295496626	0.93309119896015	-0.87055103784852
H	0.85125418127386	0.27564183297007	2.42203253614437
H	1.64742398037582	2.16124346476661	-0.29831386850999

S5. Example Orca Input Files

S5.1. Example input file for geometry optimization.

```
! B3LYP RIJCOSX def2-TZVP def2/J D3BJ Opt Freq XYZFile PModel
! SlowConv TightSCF defGrid2 KDIIS
%scf
  shift shift 0.1 erroff 0 end
  damp fac 0.9 erroff 0.001 end
end
*xyzfile charge multiplicity coordinate_file.xyz
```

S5.2. Example input file for single-point energy calculations.

```
! B3LYP RIJCOSX def2-TZVPP def2/J D3BJ PModel
! SlowConv defGrid2 KDIIS
%scf
  shift shift 0.1 erroff 0 end
  damp fac 0.9 erroff 0.001 end
end
*xyzfile charge multiplicity optimized_coordinates.xyz
%output
  Print [P_Hirshfeld] 1
end
```

S5.3. Example Orca input file for X-ray emission spectra simulations.

```
! B3LYP RIJCOSX ZORA ZORA-def2-TZVPP SARC/J D3BJ PModel
! SlowConv VeryTightSCF defGrid3 KDIIS
%basis
  newGTO Mo "old-ZORA-TZVPP" end
end
%scf
  shift shift 0.1 erroff 0 end
  damp fac 0.9 erroff 0.001 end
end
*xyzfile charge multiplicity optimized_coordinates.xyz
%xes
  CoreOrb 0
  OrbOp 0
  DoQuad true
end
```

References

[1] B. Ravel, M. Newville, ATHENA, ARTEMIS, HEPHAESTUS: data analysis for X-ray absorption spectroscopy using IFEFFIT, Journal of Synchrotron Radiation, 12 (2005) 537-541.

[2] F. Neese, Software update: The ORCA program system—Version 5.0, WIREs Computational Molecular Science, 12 (2022).

[3] S. Grimme, S. Ehrlich, L. Goerigk, Effect of the damping function in dispersion corrected density functional theory, Journal of Computational Chemistry, 32 (2011) 1456-1465.

[4] S. Grimme, J. Antony, S. Ehrlich, H. Krieg, A consistent and accurate *ab initio* parametrization of density functional dispersion correction (DFT-D) for the 94 elements H-Pu, The Journal of Chemical Physics, 132 (2010) 154104.

[5] F. Weigend, Accurate Coulomb-fitting basis sets for H to Rn, Physical Chemistry Chemical Physics, 8 (2006) 1057.

[6] D.A. Pantazis, X.-Y. Chen, C.R. Landis, F. Neese, All-electron scalar relativistic basis sets for third-row transition metal atoms, Journal of Chemical Theory and Computation, 4 (2008) 908-919.

[7] B. Helmich-Paris, B. De Souza, F. Neese, R. Izsák, An improved chain of spheres for exchange algorithm, The Journal of Chemical Physics, 155 (2021) 104109.

[8] F. Weigend, R. Ahlrichs, Balanced basis sets of split valence, triple zeta valence and quadruple zeta valence quality for H to Rn: Design and assessment of accuracy, Physical Chemistry Chemical Physics, 7 (2005) 3297.
